# FGF21‐Mediated Upregulation of SIRT1 Delays Intervertebral Disc Degeneration by Promoting PINK1/Parkin Dependent Mitophagy Through Deacetylation of FOXO3

**DOI:** 10.1111/acel.70449

**Published:** 2026-03-20

**Authors:** Zuo‐long Wu, Rui Ran, Qi‐qi Xie, Cong Zhang, Ya‐jun Chen, Peng Cheng, Ke‐Ping Wang, Hai‐hong Zhang

**Affiliations:** ^1^ Department of Orthopedics Lanzhou University Second Hospital Lanzhou China; ^2^ The Second Clinical Medical College Lanzhou University Lanzhou China; ^3^ Key Laboratory of Orthopedics Disease of Gansu Province Lanzhou University Second Hospital Lanzhou China; ^4^ Medical Sciences Program, Indiana University School of Medicine Bloomington Indiana USA

**Keywords:** cellular senescence, FGF21, FOXO3, intervertebral disc degeneration, mitophagy, SIRT1

## Abstract

Intervertebral Disc Degeneration (IDD) is a common degenerative spinal disease and a leading cause of low back pain and disability. The senescence of nucleus pulposus cells (NPCs) is a central mechanism driving the pathological progression of IDD, though its regulatory mechanisms remain unclear. Bioinformatic analysis identified FGF21 as a key gene regulating NPCs senescence. In both human and rat degenerated intervertebral discs, FGF21 expression was significantly downregulated and closely associated with the upregulation of senescence markers (P16, P21, and P53) and clinical pathological features (age, symptom duration, and Pfirrmann grading). In vitro experiments demonstrated that FGF21 intervention significantly alleviated tert‐butyl hydroperoxide (TBHP)‐induced NPCs senescence and mitochondrial damage. Mechanistically, FGF21 upregulated SIRT1 and promoted the deacetylation of FOXO3 at lysine sites K241, K258, K289, and K568, thereby enhancing mitophagy and inhibiting NPCs senescence. In vivo, FGF21 treatment significantly improved disc height and histological scores in a rat IDD model, whereas SIRT1 knockdown attenuated these protective effects. In summary, FGF21 inhibits NPCs senescence and delays IDD progression by activating SIRT1‐mediated FOXO3 deacetylation and enhancing PINK1‐Parkin pathway‐dependent mitophagy. Therefore, the FGF21‐targeted SIRT1/FOXO3/PINK1/Parkin axis may represent a promising new therapeutic strategy for IDD.

## Graphical abstract

FGF21 upregulates SIRT1 to promote the deacetylation of FOXO3 at sites K241, K258, K289, and K568, thereby activating PINK1/Parkin‐dependent mitophagy, suppressing nucleus pulposus cell senescence, and delaying intervertebral disc degeneration.

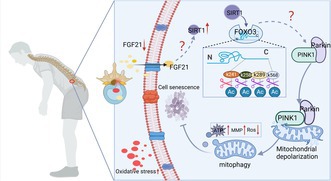

## 1 Introduction

Intervertebral Disc Degeneration (IDD) is a common degenerative disorder of the spine and a leading cause of lower back pain, which has become one of the major causes of disability worldwide (Nimgade et al. 2010; Knezevic et al. 2021). Currently, it is widely recognized that the senescence of nucleus pulposus cells (NPCs) is an intrinsic driver of IDD. Senescent NPCs exhibit reduced proliferative capacity, imbalanced extracellular matrix (ECM) metabolism, and a senescence‐associated secretory phenotype (SASP) (Song et al. 2023). These functional alterations further disrupt the homeostasis of the disc microenvironment, ultimately leading to water loss, reduced nucleus pulposus volume, and compromised biomechanical properties, thereby accelerating the progression of IDD (Wu et al. 2021). However, the precise mechanisms underlying NPCs' senescence remain unclear.

Mitophagy, a selective form of autophagy that removes damaged or dysfunctional mitochondria, plays a crucial role in maintaining cellular homeostasis by regulating energy metabolism, stress responses, and cell survival (Wu et al. 2025; Webster et al. 2014). Increasing evidence suggests that impaired mitophagy is closely associated with NPCs senescence. Specifically, mitophagy mitigates the accumulation of reactive oxygen species (ROS) and oxidative stress by clearing damaged mitochondria, thereby reducing DNA damage and inflammatory responses in cells (Kang et al. 2020; Ma et al. 2022; Gu et al. 2025). Moreover, mitophagy helps preserve mitochondrial membrane potential and ATP production, ensuring that NPCs maintain adequate energy metabolism (Wu et al. 2024). Moreover, mitophagy helps preserve mitochondrial membrane potential and ATP production, ensuring that NPCs maintain adequate energy metabolism.

Acetylation of proteins, as a dynamic and reversible post‐translational modification, regulates mitophagy by altering the conformation, stability, and activity of mitophagy‐related proteins, thereby achieving precise control over this process (Webster et al. 2014; Wang, Qi, et al. 2020). For example, Mitofusin 2 (MFN2), a key protein involved in mitochondrial outer membrane fusion, is acetylated, and this modification affects mitochondrial membrane depolarization, leading to impaired mitophagy (Alka et al. 2023). On the other hand, the acetylation of Parkin activates mitophagy and inhibits cervical cancer cell proliferation (Sun et al. 2022). The acetylation level of FOXO3 increases significantly in response to inflammatory mediators (Jiang et al. 2023) and oxidative stress (Tseng et al. 2013), whereas its deacetylation activates the PINK1/Parkin ubiquitin‐dependent mitophagy, accelerating the clearance of damaged mitochondria (Wei et al. 2024). Additionally, deacetylated FOXO3 can directly regulate the expression of the mitophagy core protein BNIP3, further enhancing the cell's response to mitochondrial damage (Yao et al. 2022). Studies have also shown that in IDD, acetylation of proteins such as P53 (Zhang et al. 2019) and P62 (Wang et al. 2023) increases, and reducing their acetylation levels can mitigate cellular senescence, suggesting that high levels of acetylation may be a risk factor for IDD.

SIRT1, a NAD^+^‐dependent deacetylase, plays an essential role in maintaining cellular homeostasis, antiaging processes, and regulating mitophagy (Zhang et al. 2020; Li, Liu, et al. 2023). For example, SIRT1 can regulate the activity of the PINK1/Parkin and AMPK pathways, promoting the recognition of damaged mitochondrial surface markers and accelerating the formation of mitochondrial autophagosomes, thus improving mitochondrial function (Ren et al. 2017). Furthermore, SIRT1 deacetylates autophagy‐related molecules (such as Beclin1, Atg5, Atg7, and LC3), promoting autophagy and the repair of cells and tissues in response to oxidative stress, nutrient starvation, and other adverse conditions (Deng et al. 2021; Huang et al. 2015; Lee et al. 2008). In IDD, the expression of SIRT1 decreases with aging and degeneration, whereas overexpression of SIRT1, by activating mitophagy, suppresses the senescence and death of NPCs, thereby delaying IDD (Zhang et al. 2011).

Fibroblast Growth Factor 21 (FGF21) is an endocrine factor secreted by various tissues, including the liver, adipose tissue, and muscles, and plays an essential role in regulating glucose and lipid metabolism, insulin sensitivity, and energy balance (Geng et al. 2020). Recent studies have also shown that FGF21 can improve aging. Transgenic mice overexpressing FGF21 have a significantly longer lifespan compared to wild‐type mice (Zhang et al. 2012). The loss of FGF21 function accelerates thymic aging, while FGF21 overexpression delays thymic degeneration, improves immune function, and extends lifespan (Youm et al. 2016, 2025). Aging cells exhibit reduced autophagy, which disrupts cellular homeostasis. FGF21 can promote autophagy, suppress cellular senescence and ECM degradation, reducing cartilage damage and inhibiting osteoarthritis progression (Lu et al. 2021). Additionally, FGF21 promotes mitophagy through an AMPK‐dependent pathway (Ma et al. 2024). Although there is growing evidence of FGF21's crucial biological roles in aging and various diseases, its role in IDD remains unclear.

In this study, we identified FGF21 as a key driver gene influencing nucleus pulposus cell senescence through bioinformatics and investigated its functions and molecular mechanisms in IDD both in vivo and in vitro. Our findings reveal that FGF21 delays IDD by upregulating SIRT1, which activates PINK1/Parkin‐dependent mitophagy and inhibits nucleus pulposus cell senescence. Moreover, we identified that SIRT1 promotes the deacetylation of FOXO3 at the K241, K258, K289, and K568 sites, which are crucial for FGF21‐mediated mitophagy. In conclusion, these findings suggest that FGF21 may serve as a potential therapeutic target for IDD.

## 2 Methods

### 2.1 Collection of Human Nucleus Pulposus Tissue Samples

Human nucleus pulposus (NP) tissue samples were collected from patients undergoing discectomy due to spinal disorders. Prior to surgery, all patients underwent standard preoperative examinations including X‐rays, MRI, electrocardiogram, coagulation profile, liver and kidney function tests, and screenings for infectious diseases. None of the patients had a history of tuberculosis, brucellosis, or tumors. MRI‐based Pfirrmann grading was performed independently by three experienced clinicians prior to sample collection. Each collected NP tissue was divided into three parts for subsequent PCR, Western blot, and immunohistochemical staining experiments. A total of 26 samples were collected: Grade II (*n* = 6), Grade III (*n* = 6), Grade IV (*n* = 8), and Grade V (*n* = 6). Informed consent was obtained from all patients, and the study protocol was approved by the Ethics Committee of Lanzhou University (Approval No. 2025A‐043).

### 2.2 Isolation and Culture of Rat NPCs

Male Sprague–Dawley (SD) rats weighing 120–150 g were purchased from the animal facility of Lanzhou University. The rats were euthanized by intraperitoneal injection of 2% pentobarbital sodium. After skin removal, the caudal vertebrae were harvested and disinfected in absolute ethanol for 2 min. The NP tissues were extracted and digested with 0.1 mg/mL collagenase II (Proteintech, China) for 20 min. The digested mixture was centrifuged at 1000 rpm for 5 min, and the pellet was resuspended in complete DMEM/F12 medium containing 15% fetal bovine serum and 1% antibiotics. Cells were transferred to culture flasks and incubated at 37°C in a 5% CO_2_ atmosphere. The culture medium was refreshed every 2–3 days, and cells were passaged upon reaching 90% confluence.

### 2.3 Cell Transfection

Rat NPCs were transduced with lentivirus carrying the target gene. Cells in the logarithmic growth phase were seeded into 6‐well plates, and when cell confluence reached approximately 50%, lentiviral particles (MOI = 20) were added along with 8 μg/mL Polybrene to enhance transduction efficiency. After 24 h, the medium was replaced with fresh complete medium. Cells were observed for fluorescence expression or harvested for further experiments 48–72 h post‐transduction.

### 2.4 Co‐Immunoprecipitation (Co‐IP) and Western Blot Analysis

Cells were lysed using the BeaverBeads Protein A/G immunoprecipitation magnetic bead kit (22202, Suzhou, China) according to the manufacturer's instructions. A total of 300 μg of protein lysate was incubated with 4 μg of anti‐Sirt1 antibody (CST, 8469) overnight at 4°C. The mixture was then incubated with Protein A/G magnetic beads at room temperature for 2 h, followed by thorough washing. The beads were boiled at 95°C for 5 min. Proteins were separated using SDS‐PAGE and transferred to PVDF membranes (Bio‐Millipore, Billerica, MA). Membranes were blocked with blocking buffer (Beyotime, China) for 1 h, then incubated overnight at 4°C with primary antibodies, followed by a 2‐h incubation at 4°C with secondary antibodies. Protein bands were visualized using ECL ultra‐sensitive chemiluminescence substrate (Biosharp, China).

### 2.5 Establishment of the Rat IDD Model

The experimental procedures were approved by the Animal Ethics Committee of the Second Hospital of Lanzhou University (Approval No. D2025‐013) and conducted in accordance with the “Guidelines for the Care and Use of Laboratory Animals.” Adult male SD rats (120–150 g, *n* = 24) were randomly divided into four groups: Healthy, IDD, IDD + FGF21, and IDD + FGF21 + sh‐Sirt1. Following anesthesia with 2% pentobarbital sodium (40 mg/kg, i.p.), intervertebral disc puncture was performed at the Co7/8 level using a 5 mL syringe needle. Rats were returned to their cages and maintained for 8 weeks post‐surgery. The dosage, administration method, and time (100 μg/kg/day, intraperitoneal injection, once daily, continued for 8 weeks) are based on previous studies on rFGF21 in a rat osteoarthritis model (Lu et al. 2021). During the experiment, all rats underwent routine health observations, including activity levels, fur condition, food/water intake, and behavioral patterns.

### 2.6 Statistical Analysis

All data were analyzed using GraphPad Prism 9 software. Each experiment was performed at least three times. Statistical significance between groups was assessed using Student's *t*‐test and one‐way ANOVA. Data are presented as mean ± standard deviation. All experiments were conducted with at least six independent biological replicates (*n* ≥ 6).

## 3 Results

### 3.1 FGF21 Is Downregulated During IDD

**FIGURE 1:**
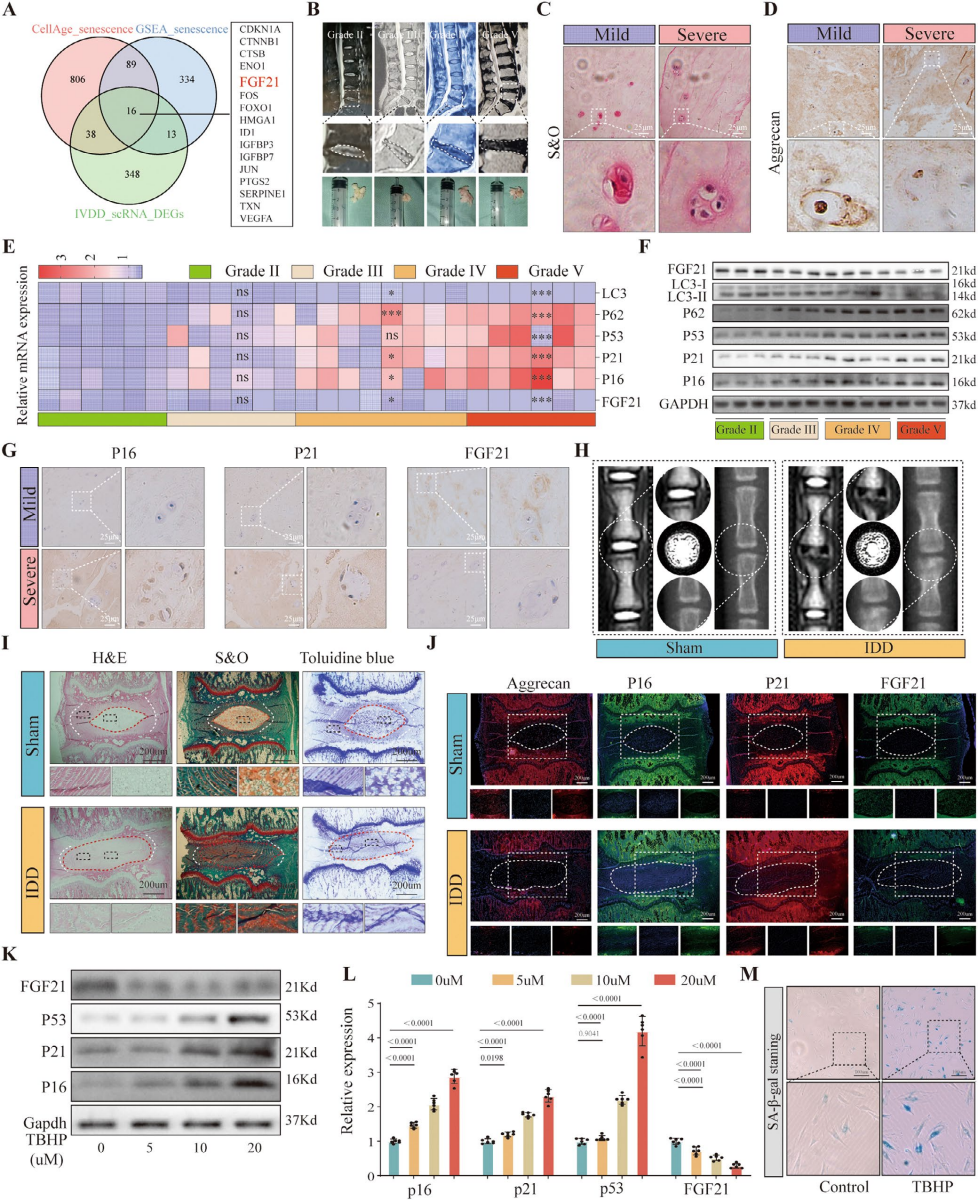
FGF21 is downregulated during IDD. (A) Venn diagram showing the overlap of differentially expressed genes (DEGs) between degenerative and non‐degenerative human NPCs with aging‐related genes from the CellAge and GSAE_Senescence_gene databases. (B) Representative T2‐weighted MRI images and corresponding intervertebral disc specimens from human samples. (C) Safranin O staining of human nucleus pulposus tissue. (D) Immunohistochemical staining of Aggrecan in human nucleus pulposus specimens. (E) Heatmap of qPCR results showing the expression of aging‐related genes in human nucleus pulposus tissues. (F) Western blot analysis of aging‐related protein expression in human nucleus pulposus tissues. (G) Immunohistochemical staining of P16, P21, and FGF21 in human nucleus pulposus samples. (H) T2‐weighted MRI and X‐ray images of rat coccygeal intervertebral discs. (I) Histological staining (H&E, Safranin O–Fast Green, and Toluidine Blue) of rat coccygeal intervertebral discs. (J) Immunofluorescence images of rat intervertebral disc sections. (K, L) Western blot analysis and quantitative bar graphs showing expression levels of aging‐related proteins in NPCs treated with increasing concentrations of TBHP. (M) Representative images of SA‐β‐Gal staining evaluating TBHP‐induced senescence in NPCs.

Nucleus pulposus cell (NPCs) senescence plays a critical role in IDD. To identify key genes involved in regulating NPCs senescence, we first analyzed single‐cell RNA sequencing datasets related to IDD from the GEO database. By intersecting DEGs between degenerated and non‐degenerated NPCs populations with senescence‐related genes from the CellAge and GSAE_Senescence_gene databases, we identified 16 senescence‐associated DEGs: CDKN1A, CTNNB1, CTSB, ENO1, FGF21, FOS, FOXO1, HMGA1, ID1, IGFBP3, IGFBP7, JUN, PTGS2, SERPINE1, TXN, and VEGFA (Figure 1A). Among them, FGF21 stood out as it has been reported to be closely linked to cellular senescence, yet its role in IDD remains unreported. Therefore, FGF21 was selected for further investigation. According to the Pfirrmann grading system, samples were categorized into mildly degenerated (Grade II–III) and severely degenerated groups (Grade IV–V) (Figure 1B). H&E staining (Figure S1A) and Safranin O staining (Figure 1C) revealed that mildly degenerated NP tissues had higher cellularity, organized collagen fibers, and abundant proteoglycans, whereas severely degenerated tissues displayed the opposite characteristics (Figure S1B). Immunohistochemical staining of Aggrecan showed reduced expression in severely degenerated discs compared to mildly degenerated ones (Figure 1D and Figure S1C). Furthermore, we evaluated the expression of senescence markers using PCR (Figure 1E), Western blotting (Figure 1F and Figure S1D), and immunohistochemistry (Figure 1G and Figure S1E–G). The results showed a progressive increase in the expression of P16, P21, and P53 with the severity of IDD, while FGF21 expression decreased correspondingly.

To further explore the clinical relevance of FGF21 in IDD, patients were stratified into high‐ and low‐FGF21 expression groups based on the median expression level. Correlation analysis with clinical parameters (age, sex, BMI, symptom duration, and Pfirrmann grade) revealed significant associations between FGF21 expression and Pfirrmann grade (*p* = 0.008), age (*p* = 0.004), and symptom duration (*p* = 0.017) (Table 1). Sankey diagram analysis demonstrated that FGF21 was highly expressed in patients younger than 65 years, with lower Pfirrmann grades (II–III), and shorter symptom durations. In contrast, patients aged ≥ 65, overweight, with higher Pfirrmann grades (IV–V), and longer symptom durations predominantly showed low FGF21 expression (Figure S2). However, logistic regression analysis showed that FGF21 is not an independent risk factor for IDD, which may be an error caused by the small sample size in this study (Table S2).

**TABLE 1:** Association between FGF21 expression and clinical characteristics of patients with IDD.

Clinical characteristics	FGF21 expression		*p*
	High expression	Low expression	
Pfirrmann	17	9	0.008**
II	6	0	
III	6	0	
IV	3	5	
V	2	4	
Sex	17	9	0.920
Female	6	3	
Male	11	6	
BMI classification	17	9	0.861
Normal	9	5	
Overweight	1	1	
Obese	6	3	
Underweight	1	0	
Age (years)	17	9	0.004**
< 65	16	4	
≥ 65	1	5	
Symptom duration (months)	17	9	0.017*
< 6	15	4	
≥ 6	2	5	

*Note:* *p < 0.05, **p < 0.01.

Based on the observed downregulation of FGF21 in human degenerative NP tissues, we established a rat IDD model via needle puncture of the coccygeal discs. Compared to the sham‐operated group, the needle‐puncture group showed decreased MRI signal intensity and significant disc height loss (Figure 1H and Figure S1H,I). Histological staining revealed that discs in the sham group maintained structural integrity, with well‐organized annulus fibrosus, large round nucleus pulposus (NP) tissues, and abundant proteoglycan content. In contrast, the puncture group exhibited disorganized AF structure, loss of normal NP morphology, and replacement by fibrous‐like tissue. Corresponding histological scores were significantly increased (Figure 1I and Figure S1J). Furthermore, immunofluorescence staining of rat coccygeal disc tissue showed reduced expression of Aggrecan and FGF21, and increased expression of senescence markers P16 and P21 in the NP region of the puncture group compared to the sham group (Figure 1J). These findings suggest that FGF21 is also downregulated in the rat IDD model and is closely associated with NP cell senescence.

In vitro, NPCs were treated with TBHP to simulate the degenerative process and further explore the pathological mechanisms of NPCs degeneration. Results from CCK‐8 assays (Figure S1K) and Western blot analysis (Figure 1K and Figure S1L,M) showed that TBHP reduced cell viability and increased the expression of senescence‐related markers P16, P21, and P53 in a time‐ and dose‐dependent manner, while concurrently decreasing the expression of FGF21. SA‐β‐Gal staining further confirmed that TBHP induced cellular senescence in NPCs (Figure 1L and Figure S1N). In addition, TBHP also induces mitochondrial damage in NPCs, manifested as mitochondrial swelling, disappearance of mitochondrial cristae, and even vacuolization. ATP content and MMP decrease (Figure S1O–P). These findings suggest that FGF21 expression is downregulated in IDD and is closely associated with NPCs senescence.

### 3.2 FGF21 Inhibits Senescence of NPCs

**FIGURE 2:**
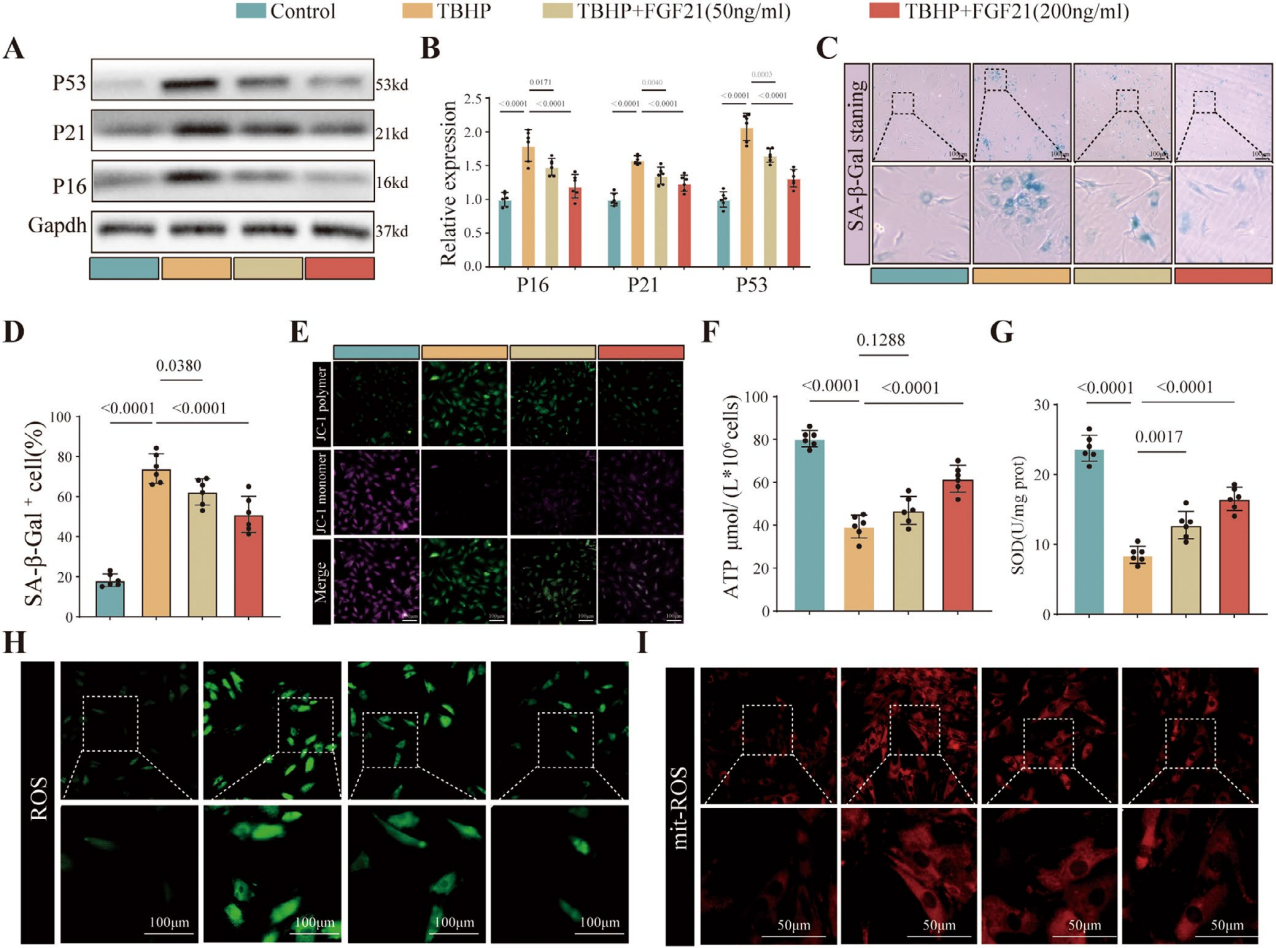
FGF21 attenuates senescence in NPCs. (A, B) Western blot analysis of senescence‐related protein expression in NPCs treated with varying concentrations of FGF21, along with corresponding quantitative bar graphs. (C, D) Representative images and quantification of SA‐β‐Gal staining showing the effect of FGF21 treatment on NPCs' senescence. (E) Fluorescence images of mitochondrial membrane potential assessed using JC‐1 staining. (F) Intracellular ATP levels measured by colorimetric assay. (G) Cellular SOD levels measured using WST‐8 assay. (H) Representative fluorescence images of intracellular reactive oxygen species (ROS) detected with ROS probes. (I) Representative images of mitochondrial ROS levels detected using MitoSOX staining. The quantitative analysis of immunofluorescence images is based on randomly selecting five fields of view from each biological replicate and taking the average, and conducting at least six independent experiments. The data points in the report represent the combined measurement values of all analyzed cells.

Since FGF21 is a secreted protein, we investigated its effect on NPCs senescence by using exogenous recombinant rat FGF21. First, a CCK‐8 assay was used to assess the impact of different concentrations of FGF21 on NPCs viability. The results showed that, in the absence of TBHP, FGF21 had no significant effect on cell viability. However, under TBHP‐induced stress, FGF21 improved cell viability in a dose‐dependent manner (Figure S3A). Based on these results, we selected 50 ng/mL (low dose) and 200 ng/mL (high dose) of recombinant rat FGF21 for further experiments. Western blot analysis revealed that FGF21 suppressed the expression of senescence‐related markers P16, P21, and P53 in a dose‐dependent manner in TBHP‐treated NPCs (Figure 2A,B). Consistent results were observed through SA‐β‐Gal staining, confirming the anti‐senescence effect of FGF21 on NPCs (Figure 2C,D). Mitochondrial dysfunction is a key hallmark of cellular senescence. We next examined whether FGF21 could modulate mitochondrial function under oxidative stress. TBHP exposure led to a reduction in mitochondrial membrane potential and ATP production in NPCs, whereas FGF21 treatment significantly restored both (Figure 2E,F and Figure S3B). Given that mitochondria are both a major source and a target of reactive oxygen species (ROS), we further evaluated the antioxidative role of FGF21. FGF21 significantly upregulated the expression of the antioxidant enzyme SOD (Figure 2G) and reduced the accumulation of MDA, a marker of lipid peroxidation (Figure S3C). Using ROS‐specific fluorescent probes, we observed that FGF21 decreased cytoplasmic ROS levels (Figure 2H and Figure S3D) and also significantly suppressed mitochondrial ROS production (Figure 2I and Figure S3E). Collectively, these findings demonstrate that FGF21 exerts a protective effect on NPCs by enhancing mitochondrial homeostasis and mitigating oxidative stress‐induced damage.

IDD is a complex pathological process driven by multiple factors, involving persistent inflammation, abnormal mechanical load, nutritional deficiency (hypoxia/hypoglycemia), and subsequent oxidative stress. In the IL‐1 β—induced degeneration model of NPCs, we also observed that FGF21 can effectively inhibit the aging of NPCs and restore IL‐1 β—induced mitochondrial damage (Figure S4).

### 3.3 FGF21 Activates Mitophagy

**FIGURE 3:**
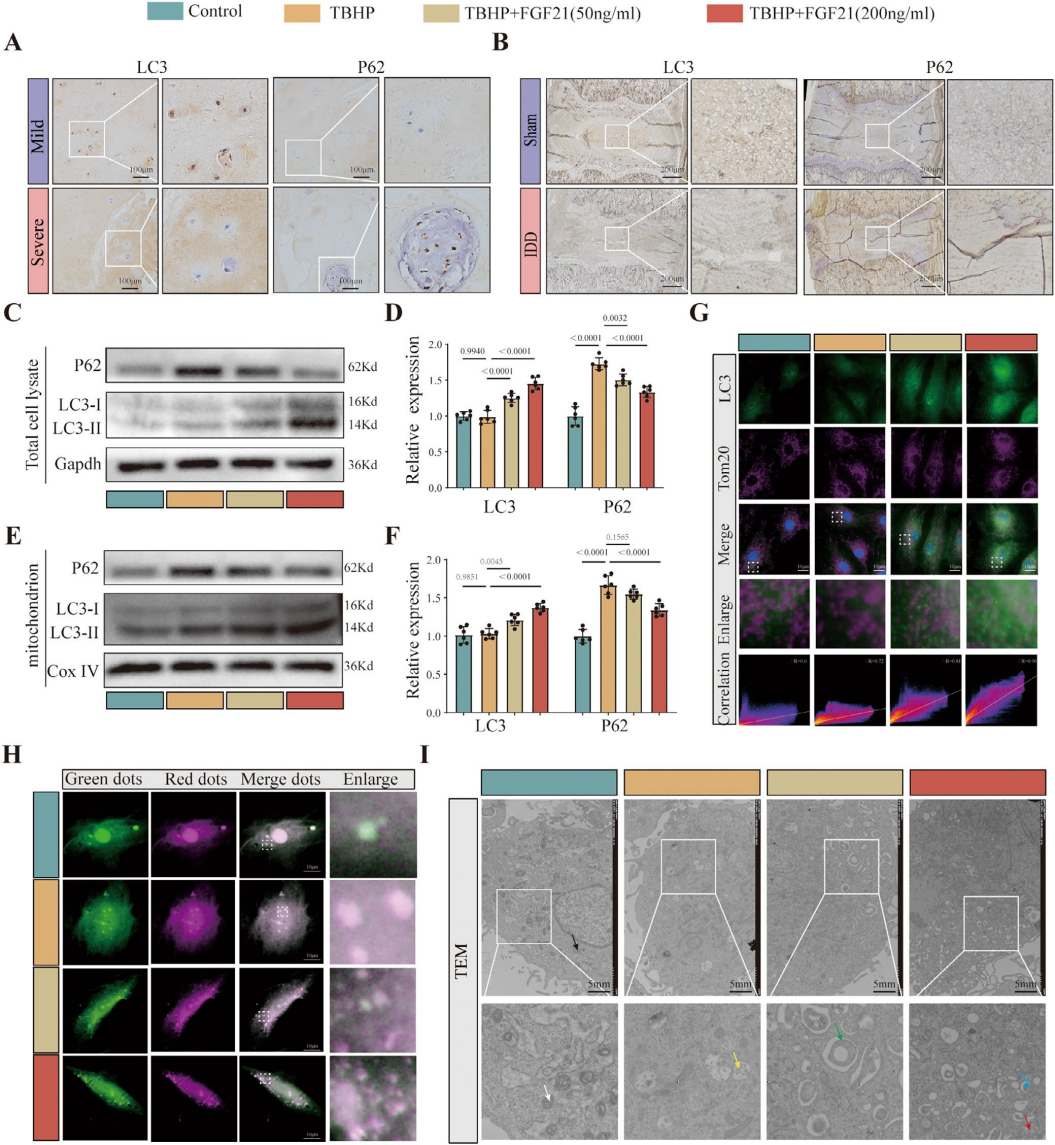
Mitophagy is impaired in IDD. (A) Immunohistochemical staining of LC3 and P62 in human NP tissues. (B) Immunohistochemical staining of LC3 and P62 in rat caudal intervertebral discs. (C, D) Western blot images of LC3 and P62 expression in whole cell lysate. (E, F) Western blot images of LC3 and P62 in mitochondrial fractions. (G) Representative confocal images showing colocalization of LC3 with mitochondrial outer membrane protein Tom20. (H) Confocal microscopy images of cells transduced with tandem mCherry‐EGFP‐LC3 virus, indicating autophagosomes (yellow puncta) and autolysosomes (red puncta). (I) Representative transmission electron microscopy (TEM) images. Black arrows indicate lysosomes, white arrows indicate mitochondria, yellow arrows indicate vacuolated mitochondria, green arrows indicate autophagosomes, blue arrows indicate autophagosome, and red arrows indicate autolysosomes.

To explore the molecular mechanism by which FGF21 counteracts senescence in NPCs, transcriptomic sequencing was performed on NPCs treated with TBHP and TBHP + FGF21 (*n* = 3). Based on the threshold of |logFC| > 1 and *p* < 0.05, a total of 398 differentially expressed genes (DEGs) were identified, including 198 upregulated and 200 downregulated genes (Table S1). Gene set enrichment analysis (GSEA) further revealed that gene expression in the FGF21‐treated group was significantly enriched in pathways related to autophagy and selective autophagy, with mitophagy being a key component (Figure S5B). These findings suggest that FGF21 may antagonize cellular senescence by activating autophagy‐related pathways. To verify this, we examined the expression of autophagy markers in human intervertebral disc specimens. Compared to mildly degenerated tissues, severely degenerated NP tissues exhibited reduced LC3 expression and elevated P62 levels (Figure 3A and Figure S7A,B). Similar results were observed in a rat IDD model: LC3II/I expression was decreased and P62 was increased in the puncture group compared to the sham group (Figure 3B and Figure S7C,D). Meanwhile, we observed the effect of TBHP on autophagy with and without the use of bafilomycin A1, and found that TBHP intervention inhibited the production of autophagy in NPCs (Figure S6A,B). Indicates that autophagy is inhibited during IDD process.

Interestingly, we observed that FGF21 not only promotes the production of autophagosomes but also their degradation, indicating that FGF21 promotes autophagic flow (Figure S6C,D). To investigate the regulatory effect of FGF21 on mitophagy, we performed Western blot analysis of autophagy‐related proteins. In the TBHP group, there was no significant change in the LC3‐II/LC3‐I ratio, while P62 accumulated in both whole‐cell lysates and mitochondrial fractions, suggesting that TBHP may inhibit mitophagy activation (Figure 3E,F). Following FGF21 treatment (50 ng/mL and 200 ng/mL), the LC3‐II/LC3‐I ratio increased in a dose‐dependent manner, accompanied by a marked reduction in P62 expression. Immunofluorescence colocalization analysis revealed enhanced colocalization of LC3 with Tom20 upon FGF21 treatment, with stronger colocalization observed in the high‐dose group (200 ng/mL) (Figure 3G). Using a tandem mCherry‐EGFP‐LC3 reporter, we found that TBHP treatment did not significantly alter autophagosome (yellow puncta) or autolysosome (red puncta) numbers. In contrast, FGF21 significantly increased both structures, indicating enhanced autophagic flux and mitophagy (Figure 3H and Figure S7E). Transmission electron microscopy further confirmed that TBHP‐treated cells exhibited swollen mitochondria with disrupted cristae and minimal autophagosomes. In contrast, FGF21‐treated cells displayed typical double‐membraned autophagosomes, with numbers increasing in a dose‐dependent manner (Figure 3I and Figure S7F). Together, these findings demonstrate that FGF21 activates mitophagy, facilitates the clearance of damaged mitochondria, and exerts a protective effect against NPCs senescence.

### 3.4 FGF21 Suppresses NPCs Senescence via Mitophagy

**FIGURE 4:**
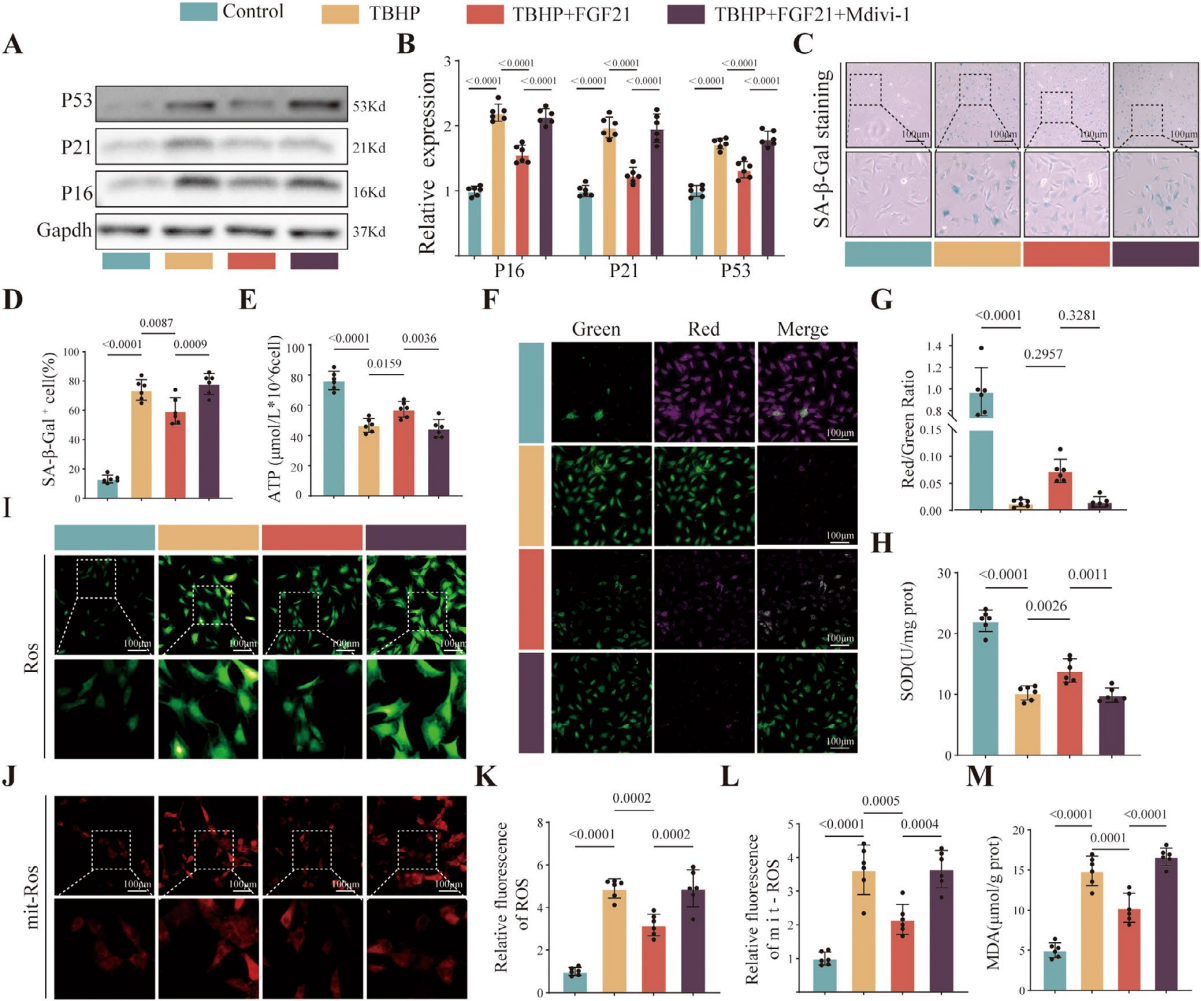
FGF21 suppresses NPCs senescence through mitophagy. (A, B) Western blot analysis and quantification of P53, P21, and P16 protein expression. (C, D) Representative images and quantification of SA‐β‐Gal staining to assess cell senescence. (E) Colorimetric assay to determine intracellular ATP levels. (F, G) JC‐1 staining for mitochondrial membrane potential and its quantification. (H) Measurement of intracellular SOD levels using WST‐8 assay. (I) Fluorescence imaging of intracellular ROS using DCFH‐DA probe. (J) Representative images of mitochondrial ROS levels assessed using Mito‐Sox probe. (K, L) Quantification of total cellular and mitochondrial ROS levels. (M) Colorimetric assay of MDA levels, a marker of lipid peroxidation. The quantitative analysis of immunofluorescence images is based on randomly selecting five fields of view from each biological replicate and taking the average, and conducting at least six independent experiments. The data points in the report represent the combined measurement values of all analyzed cells.

To investigate whether FGF21 suppresses NPCs senescence through mitophagy, the mitophagy inhibitor Mdivi‐1 was utilized. Through CKK‐18 experiment, we selected 5uM Mdivi‐1 pretreatment for 2 h, followed by co culture with FGF21 for 24 h (Figure S8A,B). The results of Western blot (Figure 4A,B) and SA‐β‐Gal staining (Figure 4C,D) showed that compared with the TBHP+FGF21 group, the inhibitory effect of FGF21 on cellular senescence was reversed when combined with the mitophagy inhibitor Mdivi‐1 (TBHP+FGF21+Mdivi‐1 group) (Figure 4C,D), indicating that the anti‐cellular senescence effect of FGF21 depends on mitophagy. The protective effect of FGF21 on mitochondrial function disappeared (Figure 4E–G). In terms of antioxidant capacity, the blockade of mitophagy weakened the ROS scavenging ability of FGF21 (Figure 4H–M), and the activation effect of FGF21 on mitophagy was also reversed after the use of Mdivi‐1 (Figure S8C–H). To eliminate off target effects of Mdivi‐1, we also used genetic methods to knock down the Drp‐1 gene. Similarly, we observed that knocking down Drp‐1 reversed the activation of mitophagy and anti‐aging effects of FGF21 (Figures S9 and S10).

### 3.5 FGF21 Activates Mitophagy via the PINK1–Parkin Pathway

To further investigate the pathway through which FGF21 activates mitophagy, we first examined the expression of key genes involved in mitophagy using qPCR. Compared to the control group, TBHP treatment increased the mRNA levels of PINK1 and Parkin. FGF21 treatment further enhanced the transcription of PINK1 and Parkin in a dose‐dependent manner, while the expression of BNIP3, BNIP3L, and FUNDC1 remained unchanged (Figure S11A). Western blot results also revealed that the expression levels of PINK1, Parkin, p‐PINK1, and p‐Parkin in the whole‐cell lysate significantly increased following TBHP intervention, and this increase was further enhanced with FGF21 treatment (Figure S11B–D). Subcellular protein fractionation experiments (Figure S11E–H) demonstrated that FGF21 not only elevated the expression of PINK1 and Parkin but also promoted their phosphorylation and mitochondrial translocation. Immunofluorescence colocalization analysis (Figure S11I) further confirmed that FGF21 increased the colocalization of Parkin with the mitochondrial membrane protein Tom20. Similarly, in an IL‐1β‐induced inflammatory model, we observed that FGF21 activated mitophagy via the PINK1‐Parkin pathway (Figure S13).

**FIGURE 5:**
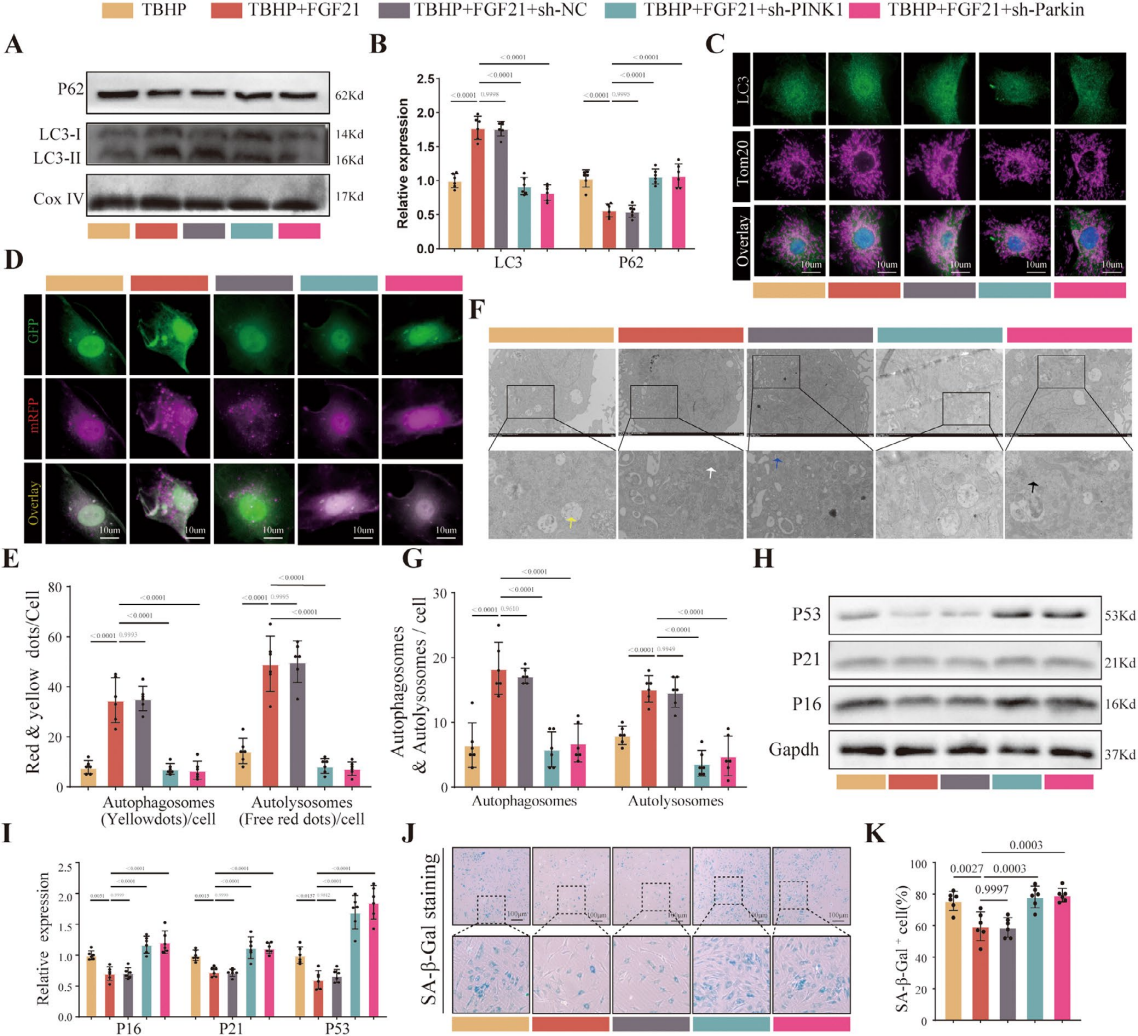
FGF21 activates the PINK1‐Parkin signaling pathway to inhibit nucleus pulposus cell senescence. (A, B) Western blot analysis of LC3 and P62 protein levels in mitochondrial fractions of cells, along with corresponding quantitative bar graphs. (C) Representative images showing colocalization of LC3 and the mitochondrial membrane protein Tom20 obtained via confocal microscopy. (D, E) Representative images and quantification bar graphs of cells infected with mCherry‐EGFP‐LC3 dual‐labeled autophagy adenovirus, observed using confocal microscopy. (F, G) Representative transmission electron microscopy (TEM) images and corresponding quantification bar graphs. (H, I) Western blot analysis and quantification of P53, P21, and P16 protein expression. (J, K) Representative images and quantification of SA‐β‐Gal staining to assess cell senescence. Black arrows indicate lysosomes, white arrows indicate mitochondria, yellow arrows indicate vacuolated mitochondria, green arrows indicate autophagosomes, blue arrows indicate autophagosome, and red arrows indicate autolysosomes. The quantitative analysis of immunofluorescence images is based on randomly selecting five fields of view from each biological replicate and taking the average, and conducting at least six independent experiments. The data points in the report represent the combined measurement values of all analyzed cells.

To investigate whether FGF21 activates mitophagy via the PINK1‐Parkin pathway, we first transfected NPCs with either a lentiviral empty vector or lentiviruses containing sh‐PINK1 and sh‐Parkin. The results showed significant suppression of both mRNA and protein expression levels of PINK1 and Parkin (Figure S12A–C), indicating the successful establishment of the gene knockdown model. Subsequent Western blot analysis of mitophagy‐related molecules revealed that, compared to the TBHP group, the TBHP+FGF21 group exhibited increased expression of LC3‐II protein in mitochondria and a decrease in P62 expression. However, when PINK1 or Parkin was knocked down, the expression levels of LC3‐II and P62 returned to those observed in the TBHP group (Figure 5A,B). Furthermore, immunofluorescence colocalization results indicated that knockdown of PINK1 or Parkin led to reduced LC3 expression and decreased colocalization (Figure 5C). Using a dual‐labeled autophagy adenovirus to monitor autophagic flux, it was observed that, compared to the TBHP group, the TBHP+FGF21 group displayed an increased number of autophagosomes (yellow puncta) and autolysosomes (red puncta). Knocking down PINK1 or Parkin counteracted the promoting effect of FGF21 (Figure 5D,E). Transmission electron microscopy results similarly demonstrated that the TBHP group exhibited mitochondrial swelling and cristae rupture, with few autophagosomes or autolysosomes present. FGF21 intervention increased autophagic structures, whereas knockdown of PINK1/Parkin reversed this effect, manifesting as increased swollen and vacuolated mitochondria and a reduction in autophagy‐related structures (Figure 5F,G). These findings collectively indicate that FGF21 activates mitophagy through the PINK1‐Parkin pathway.

We further evaluated the role of this pathway in mediating the anti‐senescent effects of FGF21. Western blot results showed that FGF21 suppressed the expression of senescence markers P16, P21, and P53 in TBHP‐treated NPCs. However, this effect was completely abolished in NPCs with PINK1 or Parkin knockdown (Figure 5H,I). Consistent results were obtained with SA‐β‐Gal staining, further indicating that the PINK1–Parkin pathway is essential for the anti‐senescent action of FGF21 (Figure 5J,K). Moreover, knockdown of PINK1 or Parkin significantly attenuated FGF21‐mediated improvements in mitochondrial function (Figure S12D–F) and antioxidant defense (Figure S12G–L).

### 3.6 FGF21 Upregulates SIRT1 Expression

**FIGURE 6:**
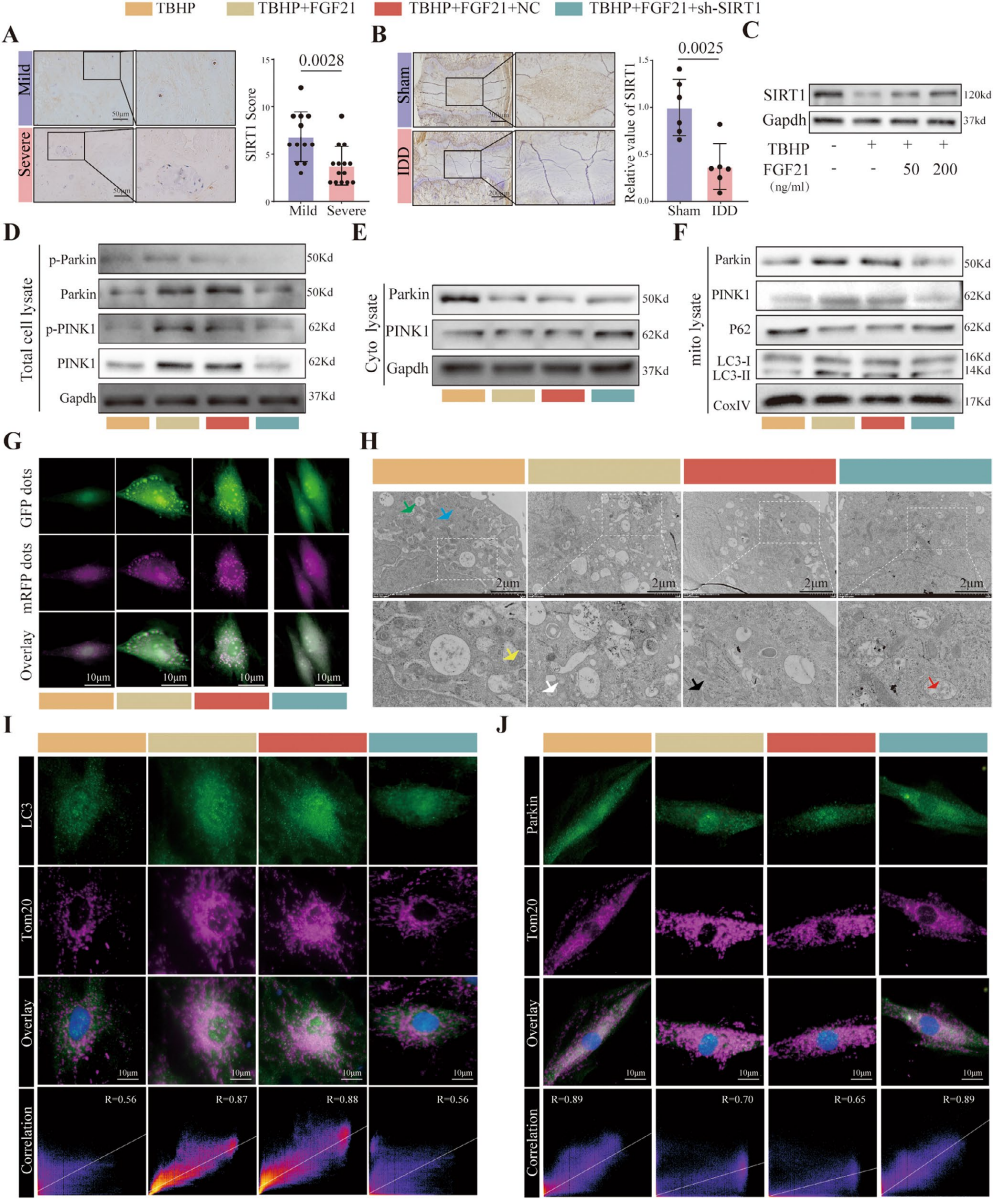
FGF21 Induces Mitophagy via SIRT1 Activation. (A) Immunohistochemical staining and quantification bar graph showing SIRT1 expression in human nucleus pulposus tissue samples. (B) Immunohistochemical staining and quantification bar graph of SIRT1 expression in rat intervertebral disc tissue samples. (C) Western blot analysis of SIRT1 protein expression. (D, E) Western blot analysis and corresponding quantification bar graphs of mitophagy‐related proteins. (F, G) Representative confocal microscopy images and quantification bar graphs of cells infected with mCherry‐EGFP‐LC3 dual‐labeled autophagy adenovirus. (H) Representative transmission electron microscopy (TEM) images. Green arrows indicate vacuolated mitochondria; blue arrows indicate lysosomes; black arrows indicate normal mitochondria; yellow arrows indicate swollen mitochondria; white arrows indicate autophagosome; red arrows indicate autolysosomes. (I) Representative immunofluorescence image showing colocalization of LC3 with Tom20. (J) Representative immunofluorescence image showing colocalization of Parkin with Tom20. (K) Representative immunofluorescence image showing colocalization of Parkin with Tom20. The quantitative analysis of immunofluorescence images is based on randomly selecting five fields of view from each biological replicate and taking the average, and conducting at least six independent experiments. The data points in the report represent the combined measurement values of all analyzed cells.

Transcriptome sequencing analysis revealed that FGF21 treatment upregulated the expression of SIRT1 (Figure S4A), suggesting that FGF21 may activate mitophagy by modulating SIRT1 levels, thereby attenuating NPCs senescence. We first assessed SIRT1 expression via immunohistochemical staining in human NP tissue samples. Compared to mildly degenerated discs, severely degenerated discs exhibited significantly reduced SIRT1 expression (Figure 6A). Consistent findings were observed in a rat model of IDD (Figure 6B). Moreover, we evaluated the regulatory effect of FGF21 on SIRT1; we observed a significant decrease in the expression level of SIRT1 in TBHP and IL1 β‐induced nucleus pulposus cell degeneration models, while the expression of SIRT1 showed a concentration dependent increase when treated with FGF21 (Figure 6C and Figure S13A,B), indicating that FGF21 effectively modulates SIRT1 expression.

### 3.7 SIRT1 Overexpression Activates Mitophagy

To explore the impact of SIRT1 on NPCs senescence and mitophagy, we examined the expression of senescence markers using Western blot and senescence‐associated staining. SIRT1 overexpression significantly suppressed TBHP‐induced NPCs senescence (Figure S14A–D). Additionally, SIRT1 overexpression improved mitochondrial function (Figure S14E–G) and reduced oxidative stress (Figure S14H–M).

We further investigated the effect of SIRT1 on PINK1/Parkin pathway‐mediated mitophagy. Western blot results showed that overexpression of SIRT1 not only increased the expression of PINK1, Parkin, p‐PINK1, and p‐Parkin in whole cell lysates but also promoted the translocation of PINK1 and Parkin from the cytoplasm to mitochondria (Figure S15A–G). Additionally, following SIRT1 overexpression, the LC3II/I ratio, the co‐localization of LC3 with the mitochondrial marker Tom20 (Figure S15H), and the co‐localization of Parkin with Tom20 were correspondingly increased, while P62 expression was reduced (Figure S15I). These results fully confirm that overexpression of SIRT1 can significantly activate PINK1/Parkin pathway‐mediated mitophagy.

### 3.8 FGF21 Inhibits NPCs Senescence by Upregulating SIRT1

To verify whether FGF21 exerts its anti‐senescence effects through SIRT1, we constructed an NPCs model with SIRT1 knockdown. qPCR and Western blot analyses revealed that the second shRNA sequence achieved the most efficient knockdown and was thus selected for subsequent experiments (Figure S16A–C). In a TBHP‐induced cellular senescence model, FGF21 treatment significantly inhibited the expression of senescence‐associated proteins P16, P21, and P53. However, this protective effect was completely abolished following SIRT1 knockdown (Figure S16D,E). β‐galactosidase staining yielded consistent results (Figure S16F,G). Moreover, FGF21 increased intracellular ATP levels and mitochondrial membrane potential (Figure S16H–J), effects that were significantly diminished in SIRT1‐deficient cells. Additionally, the antioxidant effects of FGF21 were markedly impaired upon SIRT1 knockdown (Figure S16K–M). These findings collectively demonstrate that SIRT1 is a critical downstream target mediating FGF21's anti‐senescence effects in NPCs.

### 3.9 FGF21 Activates Mitophagy Through Upregulating SIRT1

To further investigate whether FGF21 activates mitophagy through SIRT1, we examined mitophagy markers following FGF21 treatment in the presence or absence of SIRT1 knockdown. Western blot results showed that knockdown of SIRT1 not only reduced the expression of PINK1, Parkin, p‐PINK1, and p‐Parkin in whole cell lysates, but also inhibited the translocation of PINK1 and Parkin from the cytoplasm to mitochondria (Figure 6D–F and Figure S17A–D). Dynamic monitoring of autophagic flux using the mCherry‐EGFP‐LC3 dual‐fluorescence adenovirus system revealed that FGF21 treatment increased the number of autophagosomes and autolysosomes, whereas SIRT1 knockdown reduced both types of autophagic structures to levels comparable to the TBHP group (Figure 6G and Figure S17E). Transmission electron microscopy further demonstrated that in the TBHP group, mitochondria exhibited swelling, loss of cristae structure, and partial vacuolization. Following FGF21 intervention, mitochondrial swelling was alleviated, with visible contacts between autophagosomes and mitochondria, along with increased mitophagic structures. In contrast, SIRT1 knockdown significantly reduced the numbers of autophagosomes and autolysosomes (Figure 6H and Figure S17F). Furthermore, after SIRT1 knockdown, the co‐localization of LC3 with the mitochondrial membrane potential marker Tom20 (Figure 6J) and the co‐localization of Parkin with Tom20 (Figure 6K) were significantly decreased, indicating that SIRT1 plays a crucial role in FGF21‐activated mitophagy.

### 3.10 FGF21 Attenuates IDD in Rats

**FIGURE 7:**
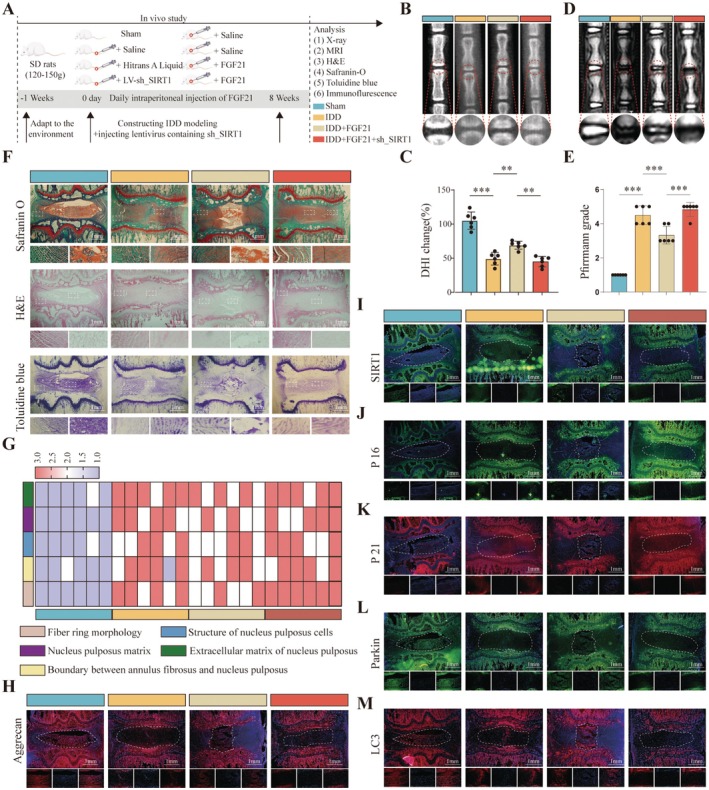
FGF21 Attenuates IDD via SIRT1 Activation. (A) Schematic diagram of the animal experimental design. (B, C) X‐ray images of rat coccygeal vertebrae and quantification bar graph of the disc height index. (D, E) MRI images of rat caudal intervertebral discs and corresponding Pfirrmann grade quantification bar graph. (F, G) Histological staining (H&E, Safranin O, and toluidine blue) of rat caudal discs and heatmap of histological scores. (H–M) Immunofluorescence staining of Aggrecan, SIRT1, P16, P21, LC3, and Parkin.

To investigate the role of FGF21 in IDD, we established a rat model of IDD and administered FGF21 via intraperitoneal injection, while also injecting sh‐SIRT1 lentivirus directly into the intervertebral disc (Figure 7A). Radiographic assessments revealed that, compared to the sham group, the IDD group exhibited a significant reduction in disc height index (DHI) and MRI signal intensity. In contrast, FGF21 treatment partially restored both DHI and MRI signal intensity. However, when SIRT1 was knocked down via sh‐SIRT1, the therapeutic effects of FGF21 were abolished (Figure 7B–E). Histological analysis further evaluated the therapeutic effects of FGF21. In the sham group, the disc structure remained intact, with large, round nucleus pulposus (NP) cells and abundant proteoglycan content. In the IDD group, the annulus fibrosus was disrupted and the NP region was largely replaced by disorganized fibrotic tissue. In the FGF21 treatment group, structural damage was partially ameliorated, evidenced by preserved NP tissue, improved annulus fibrosus morphology, increased disc height, and elevated proteoglycan levels. However, these protective effects were absent in the FGF21 + sh‐SIRT1 group, indicating that FGF21 failed to confer therapeutic benefit when SIRT1 was knocked down (Figure 7F–G). Additionally, we assessed the expression of senescence markers and mitophagy‐related proteins in the rat intervertebral discs. Immunofluorescence analysis showed that, compared to the sham group, the IDD group had significantly reduced expression of SIRT1, LC3, Parkin, and Aggrecan in the NP tissue, while levels of the senescence markers P16 and P21 were markedly elevated. FGF21 treatment reversed these changes, significantly increasing the expression of SIRT1, LC3, Parkin, and Aggrecan while decreasing P16 and P21 levels. Importantly, these beneficial effects were negated in the FGF21 + sh‐SIRT1 group (Figure 7H–M).

### 3.11 FGF21 Promotes FOXO3 Deacetylation

**FIGURE 8:**
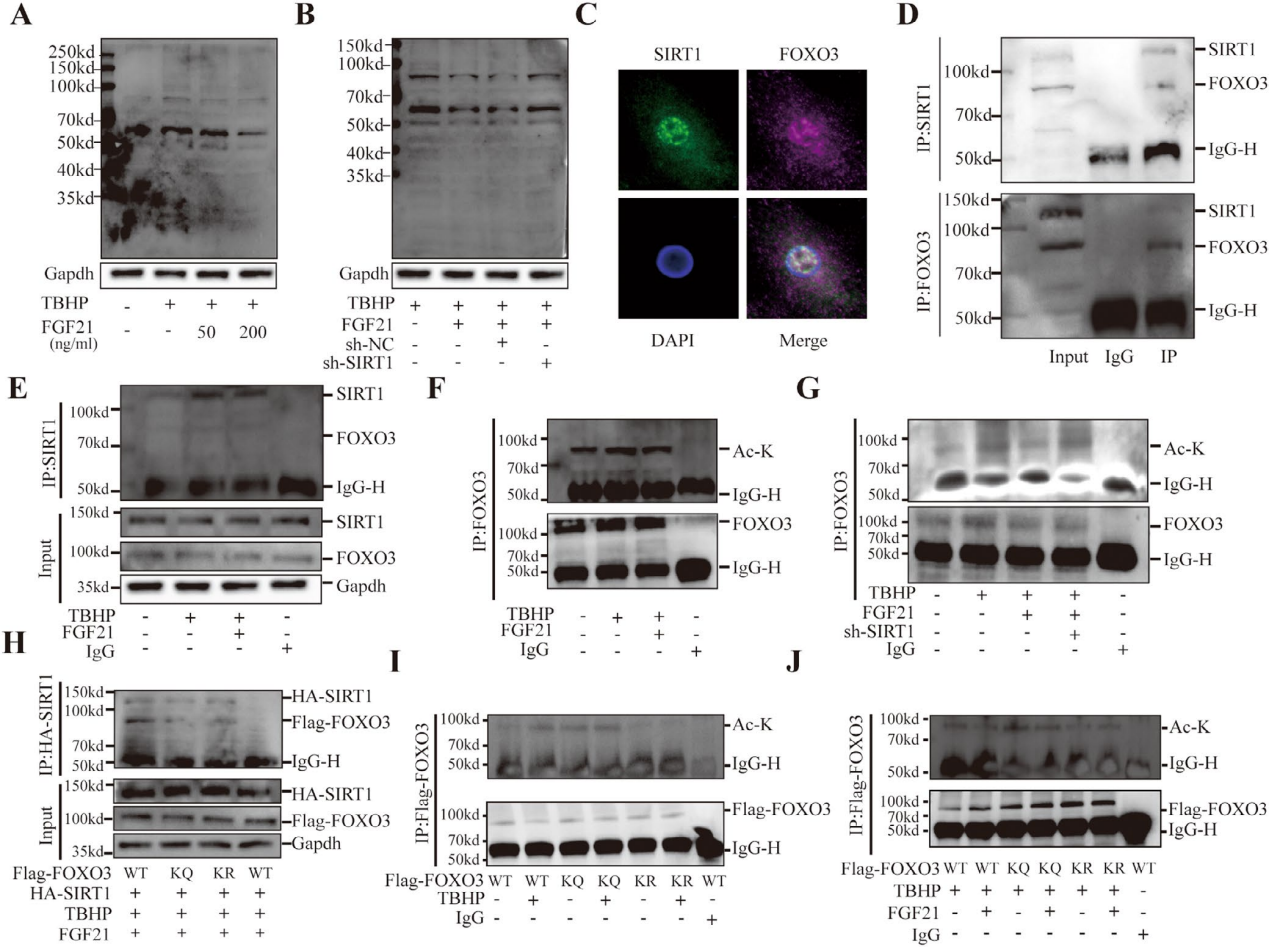
SIRT1 Regulates Deacetylation of FOXO3. (A, B) Western blot analysis of acetylation levels in whole‐cell lysates. (C) Immunofluorescence colocalization of SIRT1 and FOXO3. (D, E) Co‐immunoprecipitation (Co‐IP) showing colocalization of SIRT1 and FOXO3. (F, G) Detection of acetylation levels in FOXO3 immunoprecipitates using a pan‐acetyl‐lysine antibody. (H) Co‐IP showing the physical interaction between exogenously expressed HA‐SIRT1 and Flag‐FOXO3. (I, J) Acetylation levels of FOXO3 wild‐type (WT), FOXO3‐4KQ, and FOXO3‐4KR mutants detected using a pan‐acetyl‐lysine antibody. Ac‐K, acetylated lysine; TBHP, tert‐butyl hydroperoxide. The quantitative analysis of immunofluorescence images is based on randomly selecting five fields of view from each biological replicate and taking the average, and conducting at least six independent experiments. The data points in the report represent the combined measurement values of all analyzed cells.

To elucidate the molecular mechanism by which SIRT1 regulates mitophagy, we performed transcriptomic analysis on NPCs treated with TBHP and FGF21, with or without SIRT1 knockdown via lentiviral transduction. DEGs were enriched in pathways such as the RELA signaling pathway, TSA response pathway, GPBPN terminal protein amidation, GOCC histone acetyltransferase complex, GOMF acetylation‐dependent protein binding, and TP53 acetylation‐mediated activation (Figure S10A,B). These findings suggest that SIRT1 regulates mitophagy and cellular senescence primarily through modulating acetylation. Western blot analysis of whole‐cell lysates revealed that protein acetylation levels were significantly increased in the TBHP group compared to controls, while FGF21 treatment markedly reduced global acetylation (Figure 8A). However, this reduction was abolished upon SIRT1 knockdown (Figure 8B), indicating that SIRT1 plays a key role in FGF21‐mediated deacetylation. Notably, the most significant changes in acetylation were observed in protein bands between 50 and 70 kDa and below 100 kDa. To further identify proteins regulated by SIRT1‐mediated acetylation, we performed co‐immunoprecipitation using anti‐SIRT1 antibodies to isolate binding partners (Figure S11A,B). In total, 540 proteins were found to interact with SIRT1 (Table S2). GO showed these proteins were involved in cellular senescence, autophagy, mitophagy, and macrophage‐related functions (Figure S11C). KEGG pathway enrichment included ribosome function, glycolysis, amino acid biosynthesis, and the TCA cycle (Figure S11D). Protein–protein interaction (PPI) network analysis revealed a particularly strong association between SIRT1 and FOXO3 (Figure S11E), with FOXO3 exhibiting a relatively high number of unique peptides in the pulldown results (Figure S11F). Given the molecular weight and acetylation patterns, FOXO3 was selected as a key SIRT1 binding protein for further investigation. Immunofluorescence co‐localization analysis demonstrated clear nuclear co‐localization between SIRT1 and FOXO3 (Figure 8C). Co‐immunoprecipitation (Co‐IP) experiments confirmed the physical interaction: FOXO3 was detectable in the SIRT1 pull‐down and vice versa (Figure 8D). Further experiments revealed that while TBHP and FGF21 treatments did not alter FOXO3 protein levels, they may influence the binding interaction between SIRT1 and FOXO3 (Figure 8E). Acetylation analysis of FOXO3 IP showed increased acetylation following TBHP treatment, whereas FGF21 reduced FOXO3 acetylation. This deacetylating effect was reversed when SIRT1 was knocked down (Figure 8F,G), suggesting that FGF21 regulates FOXO3 acetylation via upregulation of SIRT1. Previous studies have identified four critical SIRT1‐targeted acetylation sites on FOXO3 in rats: K241, K258, K289, and K568 (Brunet et al. 2004; Li et al. 2017; Wang, Wei, et al. 2020). Sequence alignment demonstrated that these lysine residues are highly conserved across multiple species (Figure S11G). To further investigate their functional significance, we generated FOXO3 mutants by substituting Lys (K) with Gln (Q) to mimic acetylation (FOXO3‐4KQ) and with Arg (R) to mimic deacetylation (FOXO3‐4KR). These mutations did not disrupt the physical interaction between FOXO3 and SIRT1 (Figure 8H). TBHP treatment increased the acetylation of wild‐type FOXO3, but not of the FOXO3‐4KQ or FOXO3‐4KR mutants, indicating that K241, K258, K289, and K568 are the primary acetylation sites under oxidative stress, and that FGF21 suppresses acetylation at these critical residues (Figure 8I,J).

### 3.12 Deacetylation of FOXO3 by SIRT1 Is Essential for FGF21‐Induced Mitophagy

**FIGURE 9:**
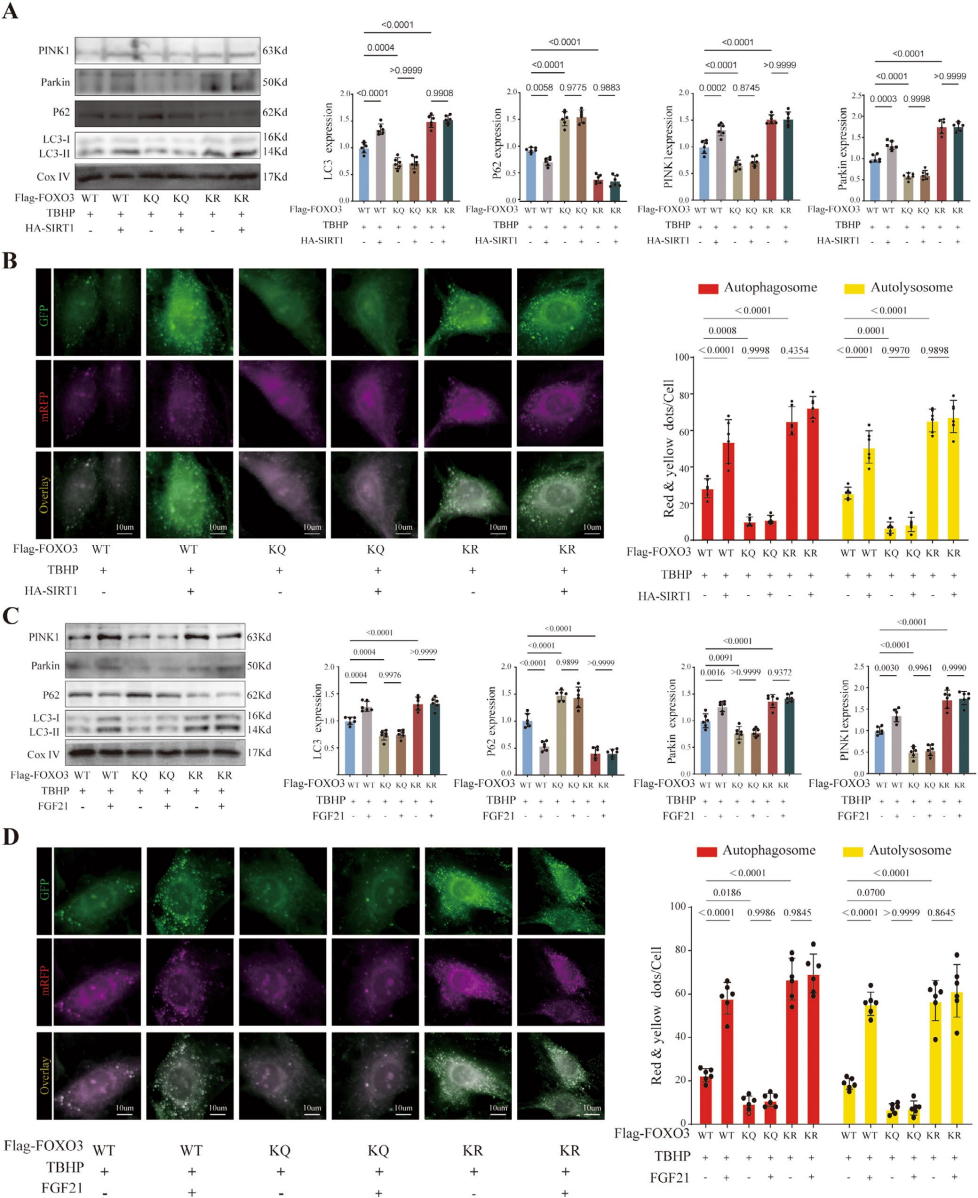
Deacetylation of FOXO3 is essential for FGF21‐induced mitophagy in NPCs. (A) Representative Western blot images and quantification bar graphs of PINK1, Parkin, LC3‐II, and P62 expression in NPCs with or without SIRT1 overexpression. (B) Confocal live‐cell imaging of mitophagy in NPCs with or without SIRT1 overexpression. Red puncta indicate autophagosomes and autolysosomes; quantification shown in bar graphs. (C) Representative Western blot images and quantification bar graphs of PINK1, Parkin, LC3‐II, and P62 expression in NPCs treated with or without FGF21. (D) Confocal live‐cell imaging of mitophagy in NPCs treated with or without FGF21, with quantification shown in bar graphs. The quantitative analysis of immunofluorescence images is based on randomly selecting five fields of view from each biological replicate and taking the average, and conducting at least six independent experiments. The data points in the report represent the combined measurement values of all analyzed cells.

To investigate the role of SIRT1‐mediated deacetylation of FOXO3 (at sites K241, K258, K289, and K568) in FGF21‐promoted mitophagy and cellular senescence, we performed validation experiments using Western blot, the mCherry‐EGFP‐LC3 dual‐fluorescence adenovirus system, senescence staining, and other assays. The results showed that transfection with acetylation‐mimicking Flag‐FOXO3 (4KQ) suppressed mitophagy and exacerbated nucleus pulposus cell senescence, whereas transfection with deacetylation‐mimicking Flag‐FOXO3 (4KR) activated mitophagy and inhibited nucleus pulposus cell senescence. Overexpression of HA‐SIRT1 enhanced mitophagy mediated by wild‐type Flag‐FOXO3 (WT) and delayed nucleus pulposus cell senescence. However, in NPCs stably expressing either Flag‐FOXO3 (4KQ) or Flag‐FOXO3 (4KR), HA‐SIRT1 overexpression did not produce the aforementioned effects (Figure 9A,B and Figure S20A,B). This indicates that the deacetylation of FOXO3 at K241, K258, K289, and K568 occurs downstream of SIRT1, and that acetylation modifications at these sites play a critical regulatory role in mitophagy and nucleus pulposus cell senescence.

Furthermore, we investigated the effects of FGF21 on mitophagy and nucleus pulposus cell senescence following mutation of the aforementioned FOXO3 sites. The experimental results showed that FGF21 treatment produced effects similar to those of HA‐SIRT1 overexpression, promoting mitophagy and inhibiting cellular senescence (Figure 9C,D and Figure S20C,D). However, when SIRT1 was knocked down simultaneously with FGF21 treatment, the activation of mitophagy and the inhibition of senescence by FGF21 were blocked in the context of wild‐type FOXO3. In contrast, in cells expressing acetylation‐mimicking (KQ) or deacetylation‐mimicking (KR) mutant forms, SIRT1 knockdown did not affect the phenotypes of mitophagy and cellular senescence (Figures S21 and S22).

In summary, these results collectively demonstrate that SIRT1‐mediated deacetylation of FOXO3 is a necessary step for FGF21 to activate mitophagy and inhibit nucleus pulposus cell senescence.

## 4 Discussion

In this study, we identified FGF21 as a key driver gene involved in the regulation of nucleus pulposus cell (NPCs) senescence through bioinformatic analysis. We further validated its downregulation in clinical disc specimens, a rat model of IDD, and TBHP‐induced NPCs degeneration in vitro. Exogenous FGF21 intervention significantly alleviated TBHP‐induced NPCs senescence and reduced oxidative stress‐induced cellular damage, suggesting that FGF21 can inhibit oxidative stress and thereby delay NPCs senescence. These findings are consistent with previous reports. For example, vascular aging is a recognized independent risk factor for atherosclerosis, and FGF21 has been shown to protect vascular endothelial cells from H_2_O_2_‐induced premature senescence (Yan et al. 2017). Moreover, circulating FGF21 levels tend to increase under pathological conditions and positively correlate with age under physiological conditions, indicating that circulating FGF21 may serve as a key biomarker of aging (Hanks et al. 2015). These studies further support our conclusion that FGF21 plays a protective role against NPCs senescence.

To explore the underlying mechanisms by which FGF21 exerts its anti‐senescence effects, we performed transcriptome sequencing on NPCs subjected to TBHP‐induced stress, with or without FGF21 treatment. Functional and pathway enrichment analyses revealed that FGF21 significantly activated mitophagy. Subsequent experiments confirmed that FGF21 enhanced mitophagy in NPCs by activating the PINK1‐Parkin signaling pathway, thereby suppressing cellular senescence. Similarly, we observed that FGF21 could activate PINK1/Parkin pathway‐mediated mitophagy and inhibit nucleus pulposus cell senescence in an IL‐1β‐induced degeneration model. This indicates that the protective effects of FGF21 are consistent across different stress conditions. Our previous work showed that EGR1 knockdown activates PINK1‐Parkin‐dependent mitophagy and delays NPCs senescence (Wu et al. 2024). Similarly, Wang et al. (2018) demonstrated that PINK1 knockdown impairs mitophagy and exacerbates H₂O₂‐induced mitochondrial dysfunction and NPCs aging. Together, these findings underscore the pivotal role of PINK1‐Parkin‐dependent mitophagy in protecting NPCs from senescence, maintaining mitochondrial homeostasis, and delaying IDD progression. While some studies have suggested that Parkin expression may influence FGF21 levels (Delgado‐Anglés et al. 2024), whether FGF21 directly regulates PINK1‐Parkin‐mediated mitophagy had remained unclear. Our study is the first to elucidate this regulatory mechanism, offering a novel perspective on the role of FGF21 in IDD and laying a theoretical foundation for future research.

Interestingly, we observed a paradoxical phenomenon: under TBHP‐induced oxidative stress, NPCs exhibited increased expression of PINK1 and Parkin, yet mitophagic activity was suppressed. This discrepancy may represent a compensatory response to oxidative stress. When mitophagy is impaired, reactive oxygen species (ROS) accumulate excessively in the cytoplasm (Li, Jiang, et al. 2023), potentially triggering upregulation of PINK1 and Parkin in an attempt to initiate mitophagy. However, despite elevated protein levels, PINK1 and Parkin were not efficiently recruited to the mitochondrial membrane, and thus mitophagy was not successfully activated. Supporting this hypothesis, subcellular protein fractionation revealed that TBHP treatment significantly increased cytosolic Parkin levels without affecting its mitochondrial localization. Previous studies have also noted that ROS are critical regulators of the PINK1‐Parkin pathway and influence mitophagy in a cell‐type‐specific manner (Xiao et al. 2017; Bingol and Sheng 2016). However, the precise mechanisms by which ROS regulate this pathway in NPCs remain unclear and warrant further investigation to elucidate potential therapeutic targets.

SIRT1 plays a central role in the regulation of mitophagy. For example, SIRT1‐deficient mice exhibit exacerbated airway resistance and lung injury upon cigarette smoke exposure, while SIRT1 overexpression protects against mitochondrial damage and cellular aging via mitophagy activation (Jiang et al. 2022). SIRT1 deficiency also leads to the accumulation of dysfunctional mitochondria, excessive mitochondrial ROS production, and cytoplasmic release of mitochondrial DNA, which in turn activates the NLRP3 inflammasome and the cytosolic nucleotide sensing pathways (STING) (Jiang et al. 2024). In our study, we found that SIRT1 acts downstream of FGF21 and promotes mitophagy by upregulating PINK1 and Parkin expression and facilitating Parkin recruitment to the mitochondrial outer membrane. Knockdown of SIRT1 abrogated the mitophagy‐promoting effects of FGF21 in NPCs. Similarly, in a rat IDD model, SIRT1 downregulation suppressed the protective effects of FGF21 on intervertebral disc integrity, indicating a critical role for SIRT1 in IDD pathogenesis.

As a major deacetylase, SIRT1 is known to deacetylate several mitophagy‐related proteins. Previous studies have shown that SIRT1‐mediated deacetylation of MFN2 at K655 and K662 promotes mitophagy and reduces ischemia–reperfusion injury in aged livers (Chun et al. 2018). Moreover, SIRT1 deacetylates SOD to modulate mitophagy and mitigate environmental stress‐induced mitochondrial damage and osteoblast senescence (Zhou et al. 2023). Our findings revealed that FGF21 upregulates SIRT1 expression and induces deacetylation of FOXO3. Specifically, deacetylation at FOXO3 lysine residues K241, K258, K289, and K568—mediated by SIRT1—is a key step in FGF21‐enhanced mitophagic flux.

This study has several limitations that warrant consideration. First, it was conducted exclusively in male Sprague–Dawley rats. Given potential sex‐based differences in disc metabolism and systemic FGF21 signaling, the generalizability of our findings to females remains uncertain and requires validation in female animal models. Second, although FGF21 administration did not induce obvious systemic adverse effects in our experiments, its known pleiotropic metabolic actions raise the possibility of indirect contributions to disc protection through improvement of systemic metabolism. Future studies incorporating longitudinal metabolic monitoring and localized delivery approaches would help distinguish between its systemic and direct local effects. Third, while the systemic FGF21 regimen used here was effective, its pharmacokinetic profile in disc tissue remains uncharacterized. Optimization of dosage, route, and frequency of administration based on tissue‐specific pharmacokinetic data would facilitate clinical translation.

**FIGURE 10:**
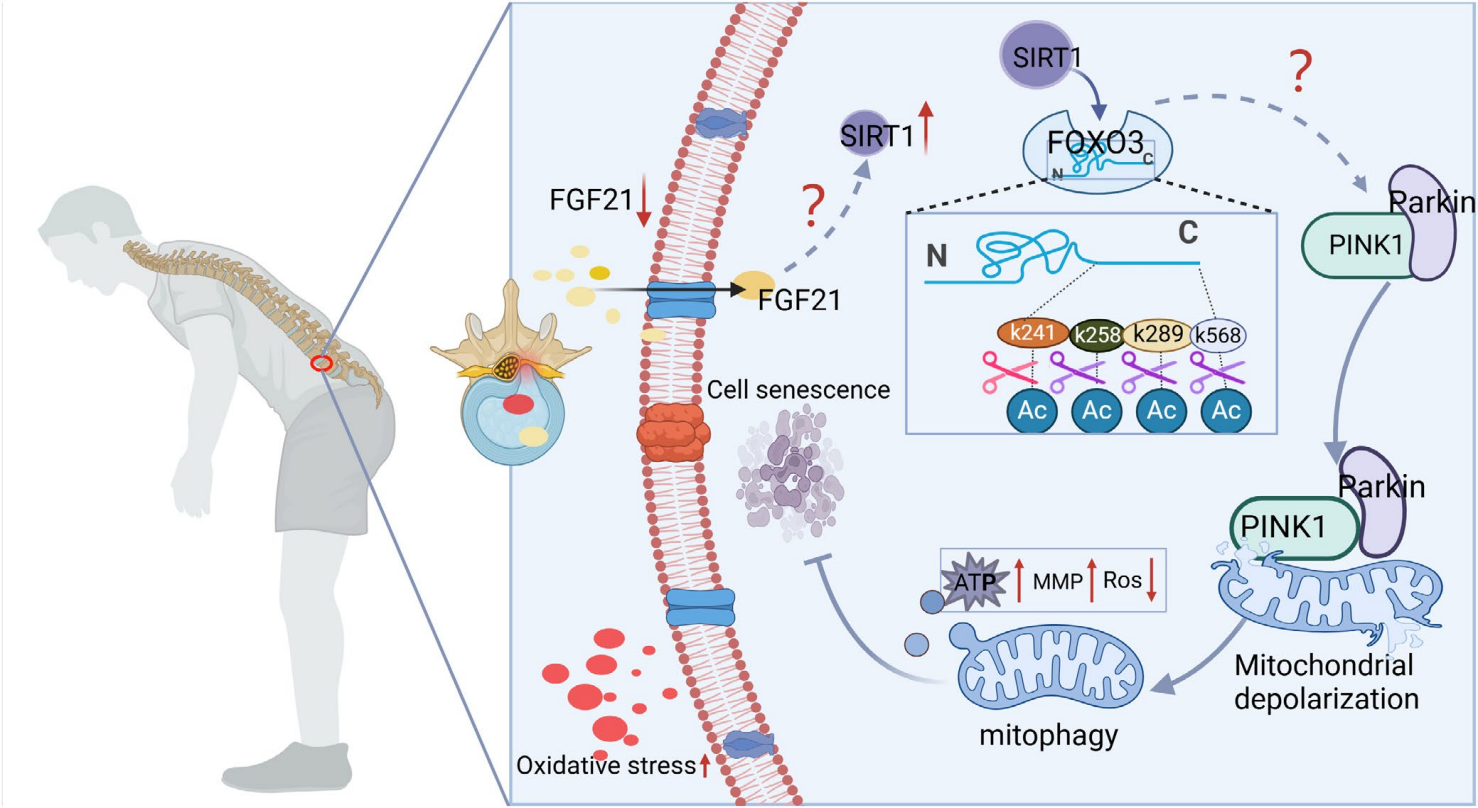
Schematic illustration of FGF21‐mediated attenuation of IDD.

In conclusion, we demonstrate that FGF21 inhibits NPCs senescence by activating mitophagy. SIRT1‐mediated deacetylation of FOXO3 at K241, K258, K289, and K568 is a critical mechanism through which FGF21 enhances mitophagic flux. This study provides theoretical support for the clinical application of FGF21 and offers valuable insights for the development of IDD therapies (Figure 10).

## Author Contributions

All authors approved the final version to be published. Zuo‐long Wu wrote the original draft of the paper. Zuo‐long Wu, Rui Ran, and Qi‐qi Xie performed the analysis of data. Ya‐jun Chen, Ke‐ping Wang, Peng Cheng, and Cong Zhang performed the acquisition of data. Zuo‐long Wu and Hai‐hong Zhang performed the study conception, design, and project administration. Zuo‐long Wu, Rui Ran, and Qi‐qi Xie are co‐first authors.

## Funding

The work was Supported by the National Natural Science Foundation of China (82360435 and 85260435). Gansu Provincial Youth Science and Technology Fund (26JRRA849) and Health Industry in Gansu Province (GSWSKY2021‐007).

## Ethics Statement

Animal trials were authorized by the Animal Ethics Committee of the Second Hospital of Lanzhou University (No. D2025–013).

## Conflicts of Interest

The authors declare no conflicts of interest.

## Supporting information


**Appendix S1:** acel70449‐sup‐0001‐AppendixS1.docx.

**Appendix S2:** acel70449‐sup‐0002‐AppendixS2.pdf.

**Table S1:** acel70449‐sup‐0003‐TableS1.xlsx.

## Data Availability

The transcriptome data generated in this study are available in the GEO database under accession number GSE316767. The CoIP/MS data have been deposited to the PRIDE database with the identifier PXD073166.
