# Supplementary material for: FGF21‐Mediated Upregulation of SIRT1 Delays Intervertebral Disc Degeneration by Promoting PINK1/Parkin Dependent Mitophagy Through Deacetylation of FOXO3
Source: Aging Cell. 2026 Mar 20;25(4):e70449. doi: 10.1111/acel.70449 (PMC13092504; doi:10.1111/acel.70449)
Supplement: Supplementary file 2 — Appendix S2: acel70449‐sup‐0002‐AppendixS2.pdf. [file ACEL-25-e70449-s003.pdf]

FIg 1F

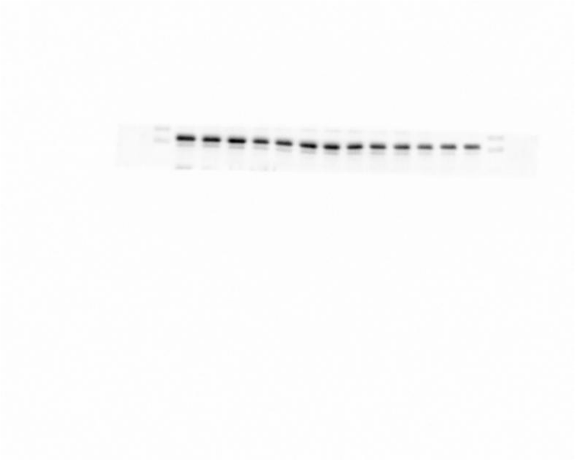

FGF21

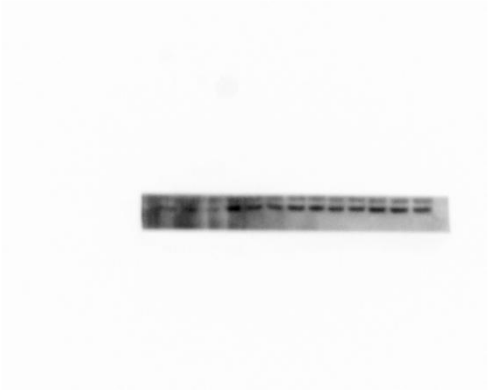

LC3

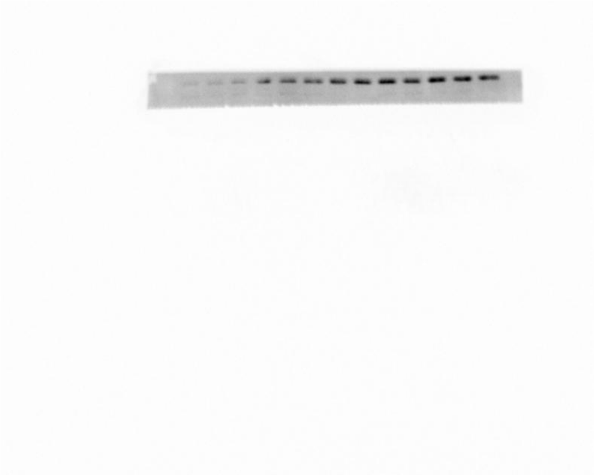

p16

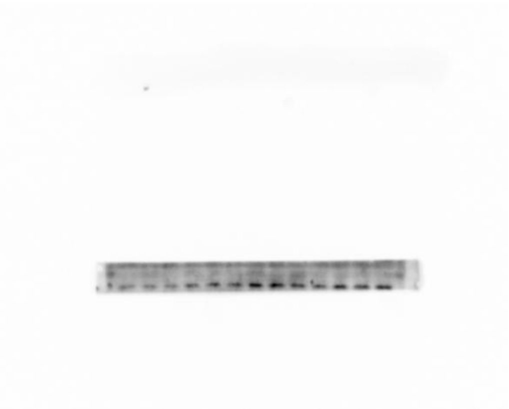

p21

P53

P62

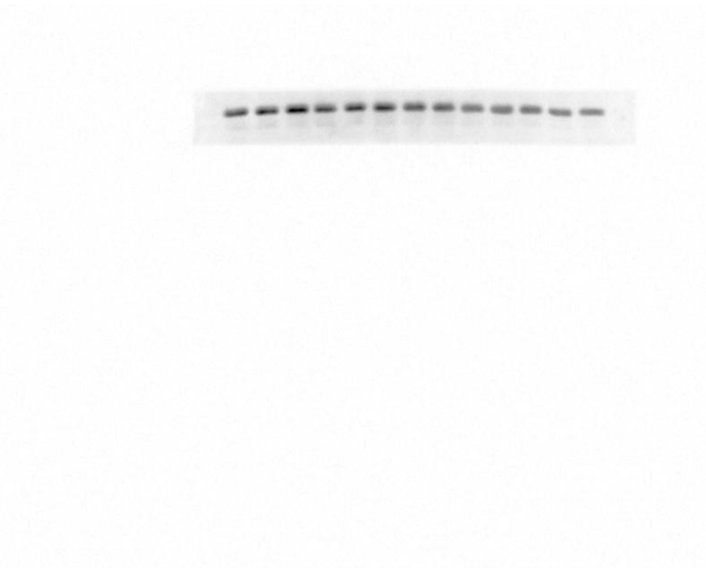

Gapdh

Fig1K

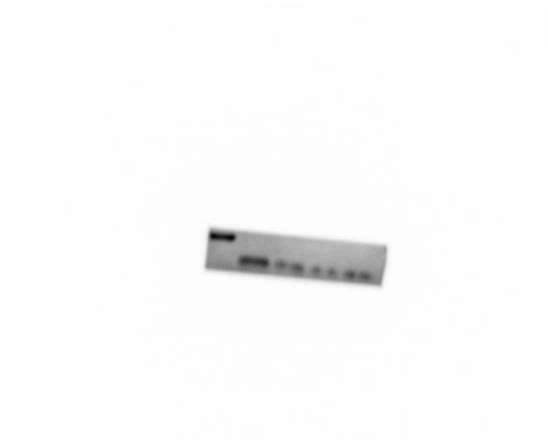

FGF21

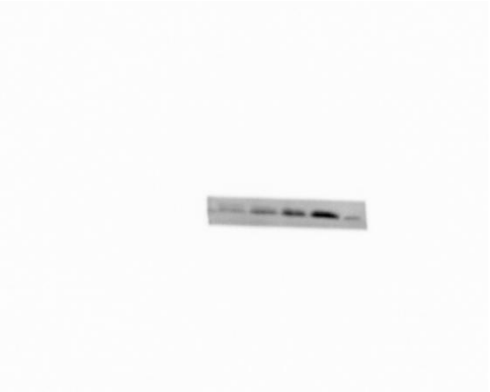

P16

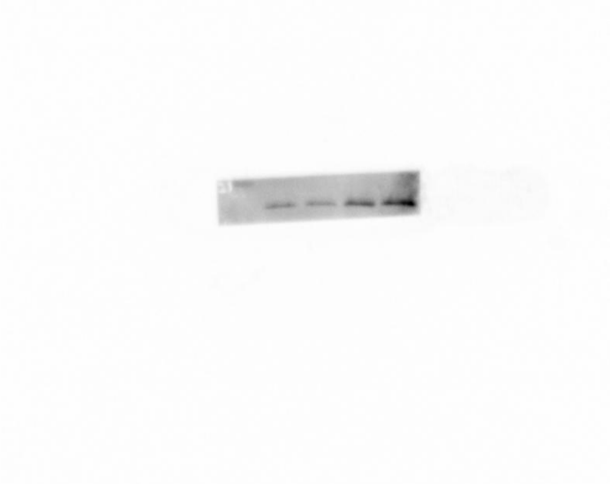

P21

p53

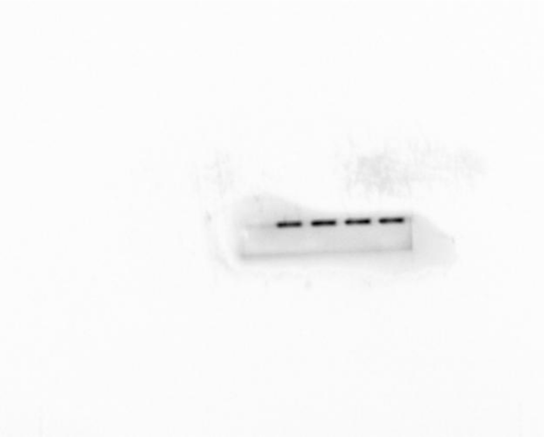

Gapdh

Fig2A

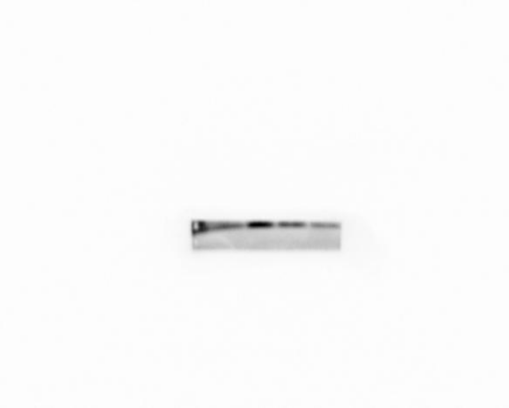

P16

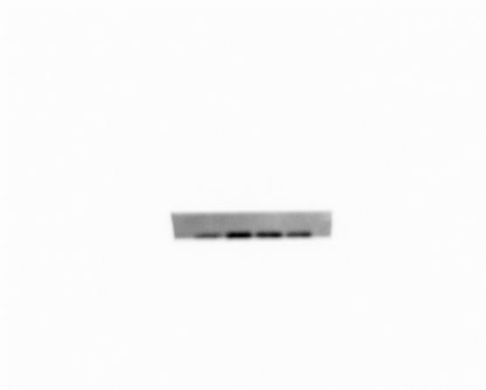

P21

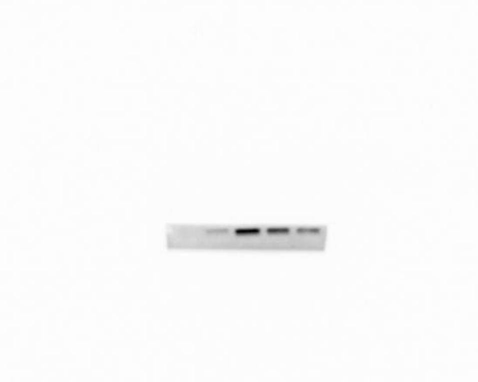

P53

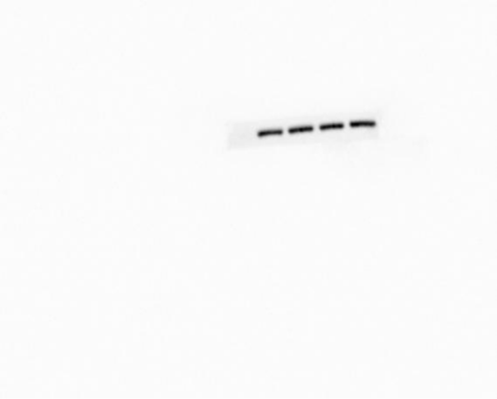

Gapdh

Fig3

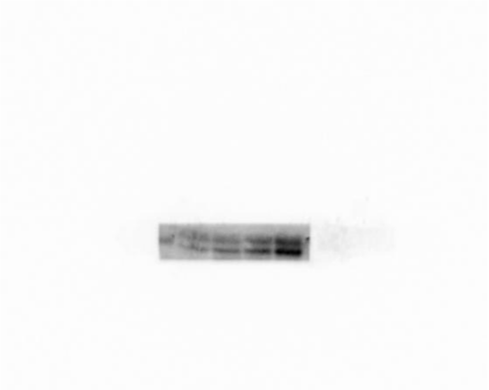

Lc3

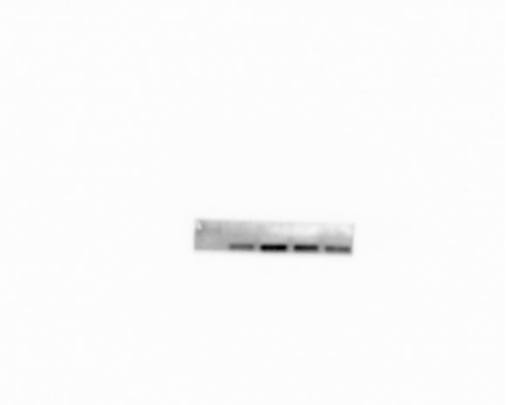

P62

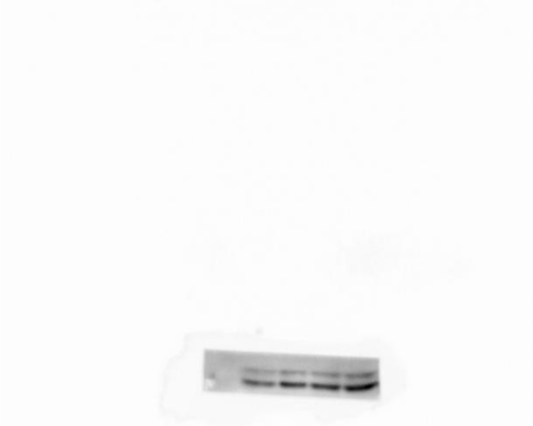

Lc3

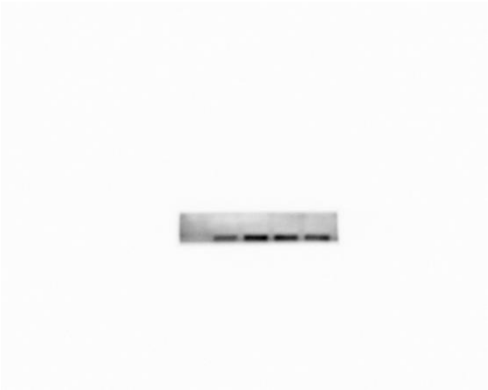

P62

Flg4

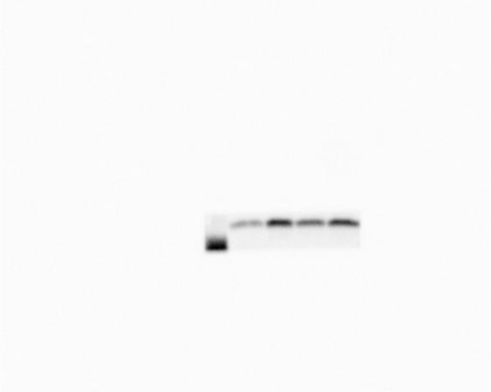

P16

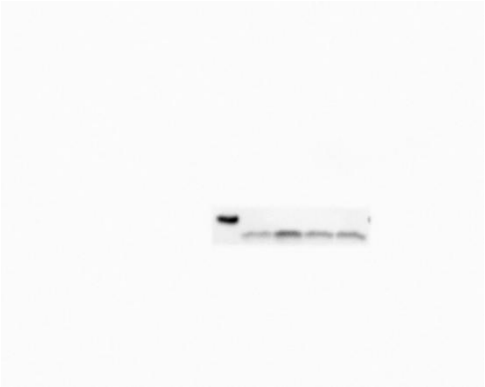

P21

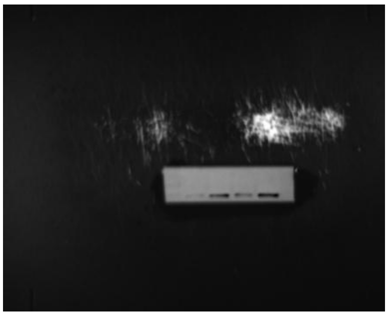

P53 Gapdh

Fig5

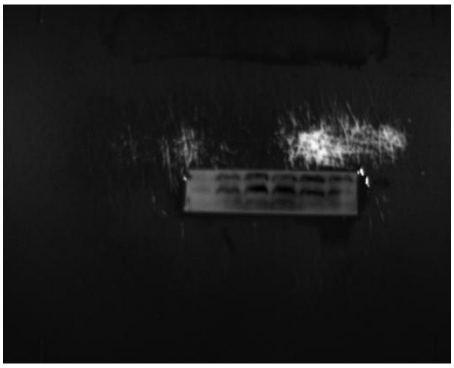

P62 Lc3

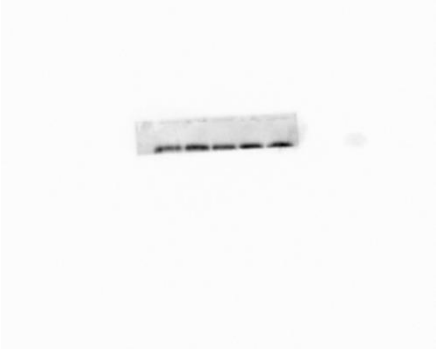

Cox IV

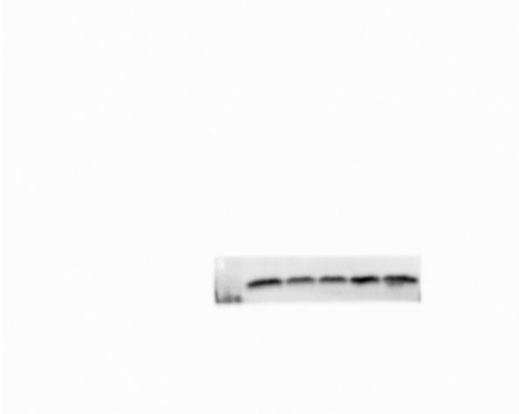

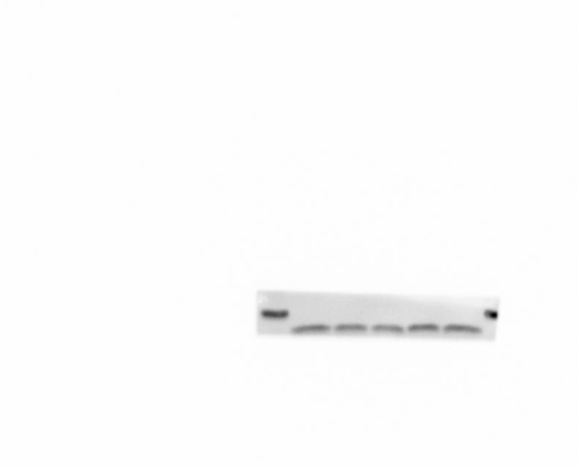

P16 p21

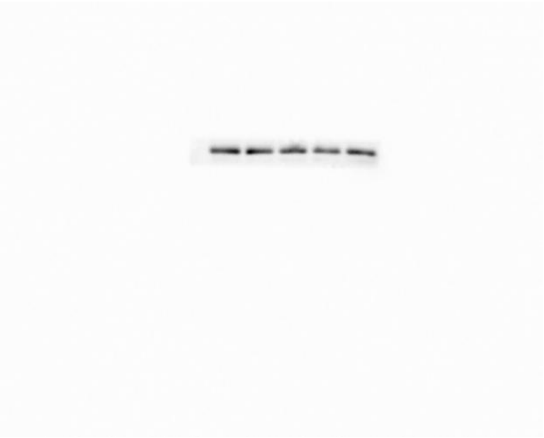

P53 Gapdh

Fig6

图 C

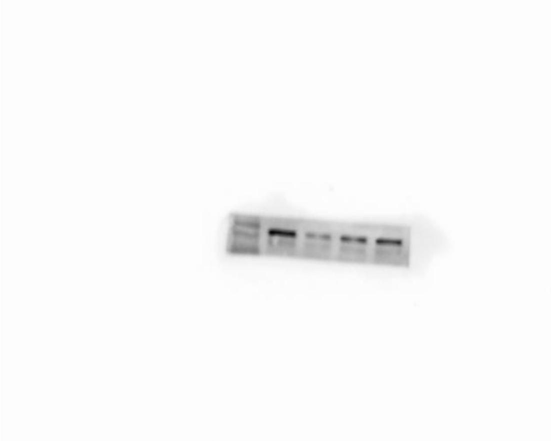

Sirt1 Gapdh

图 6D

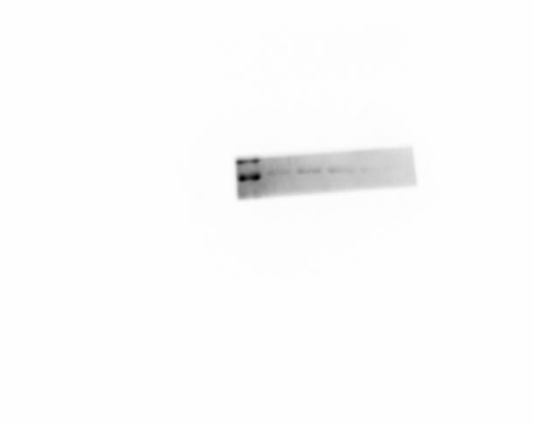

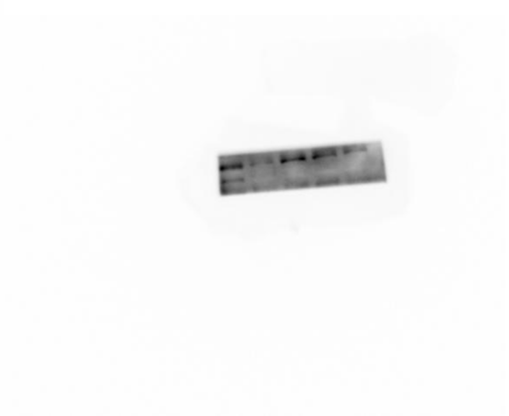

P-Parkin p-PINK1

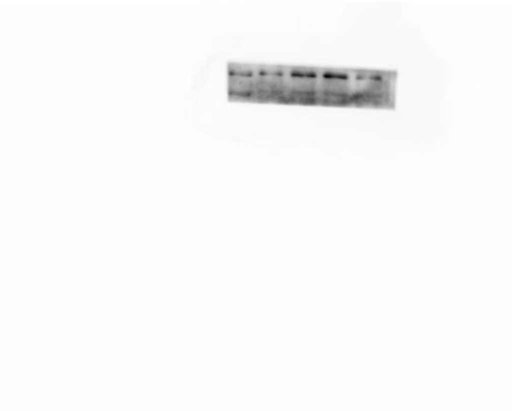

Parkin

PINK1

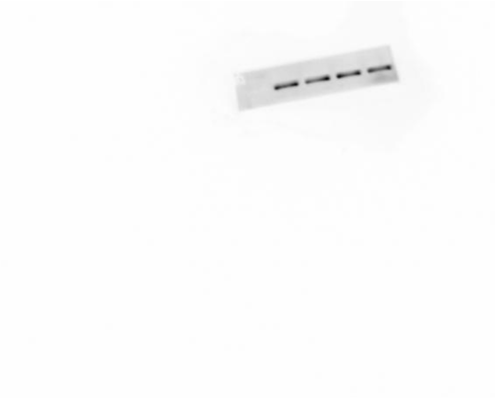

Gaodh

图 6E

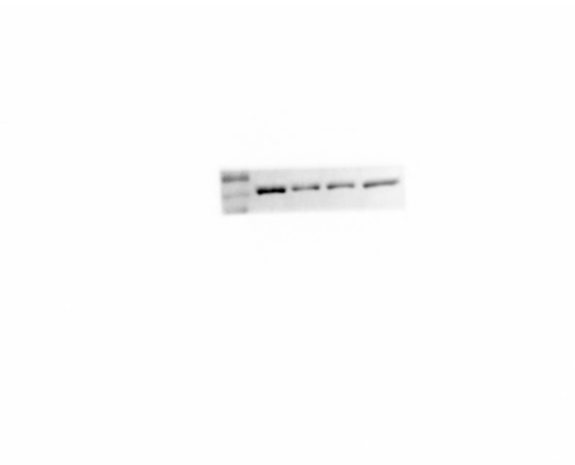

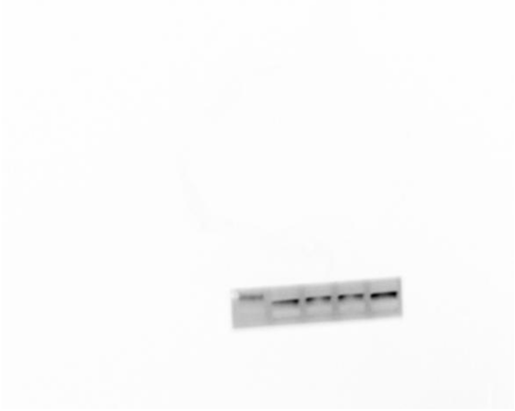

Parkin

PINK1

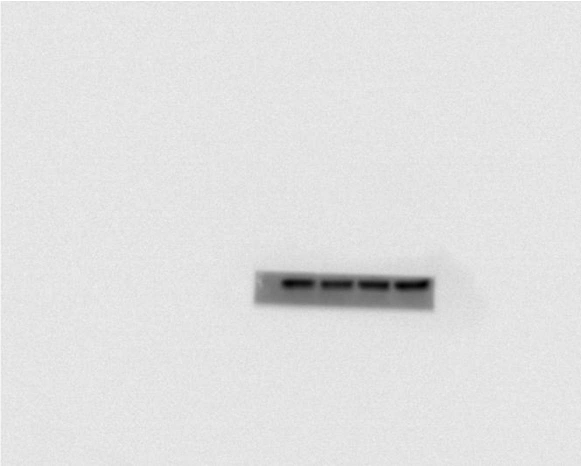

Gapdh

图 6F

Parkin

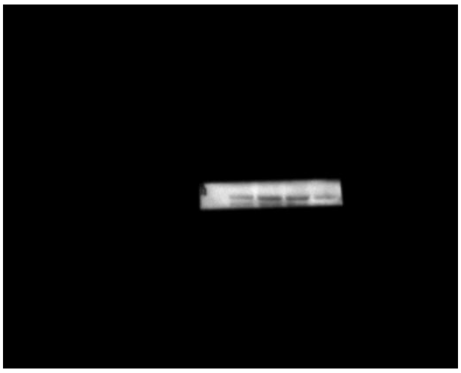

PINK1

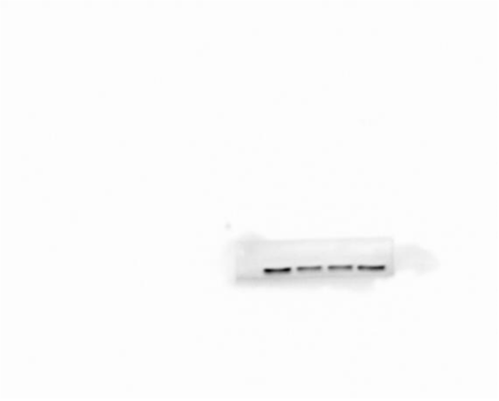

P62

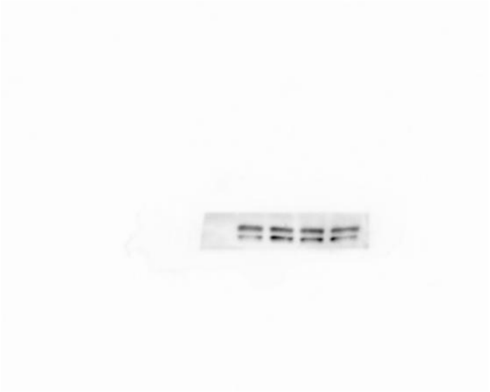

LC3

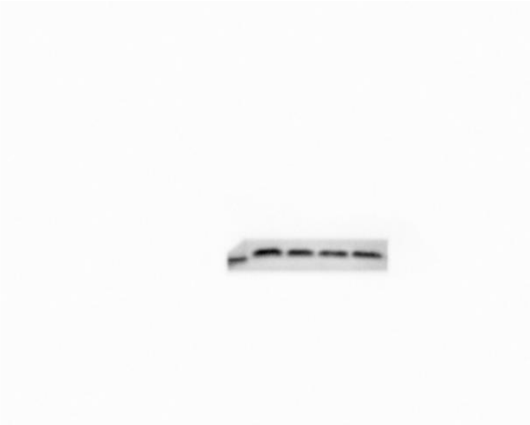

Cox IV

图 9

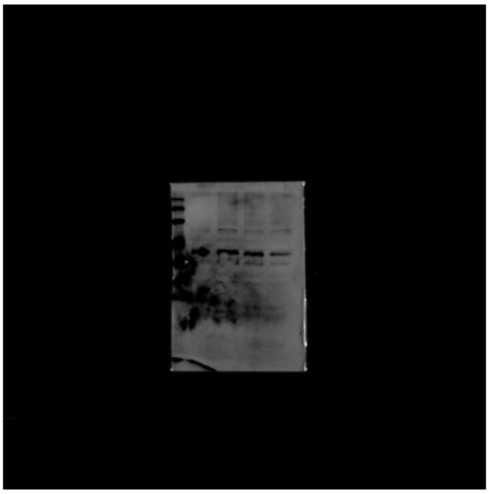

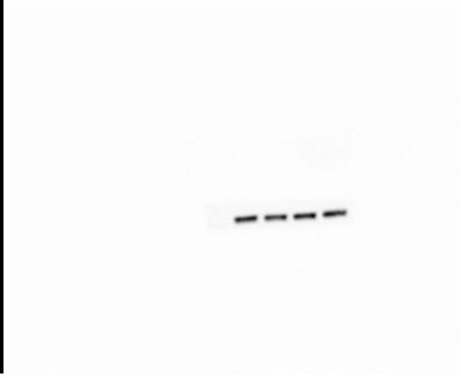

AC

gapdh

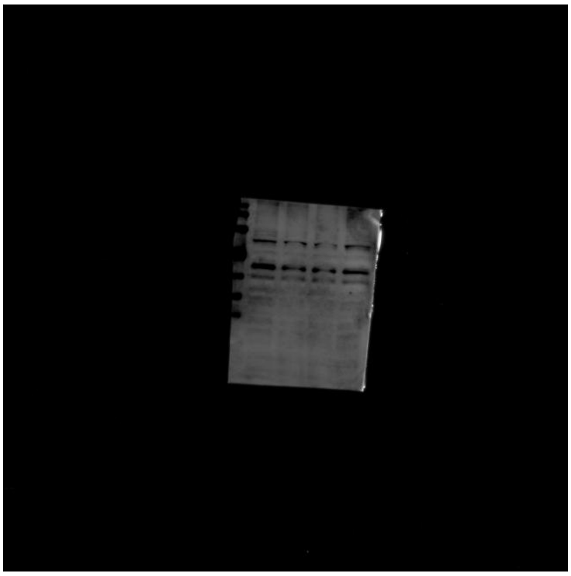

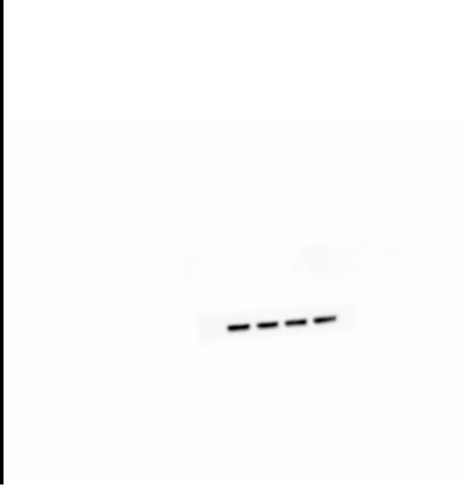

AC

Gapdh

图 D

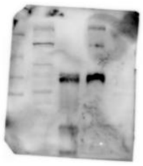

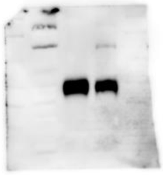

Co-IP

图 E

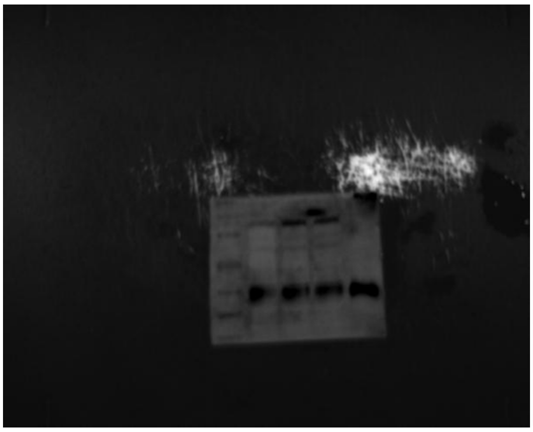

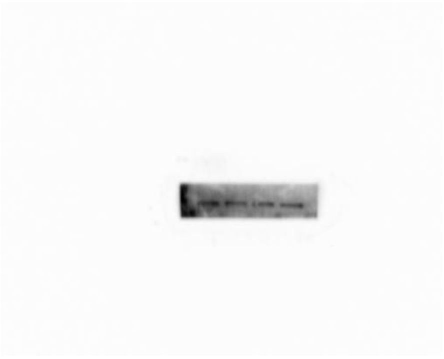

Sirt&foxo3 FOXO3

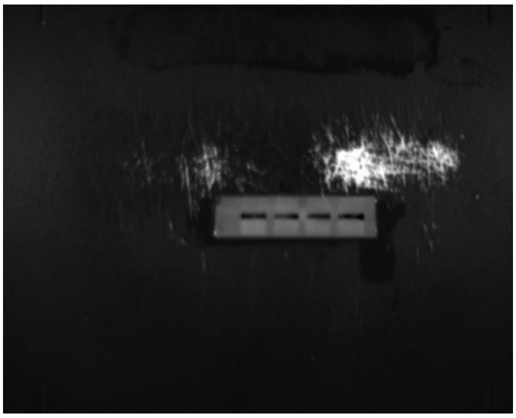

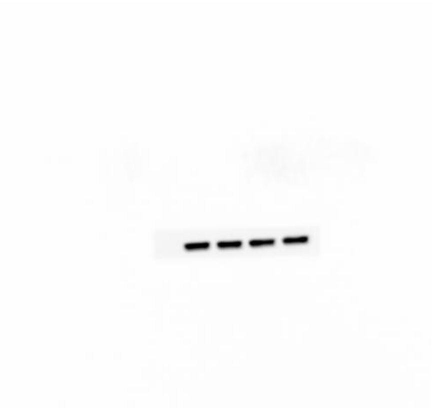

SIRT1 gapdh

图 F

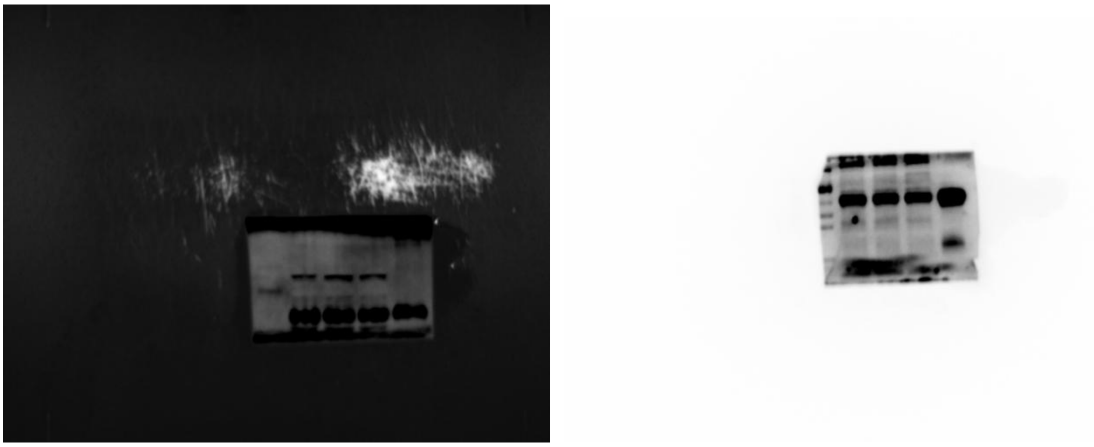

图 G

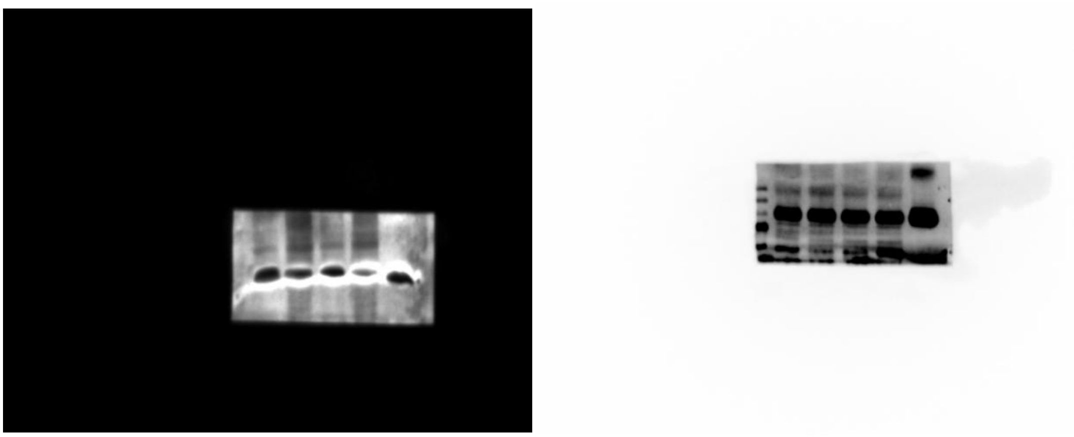

图 H

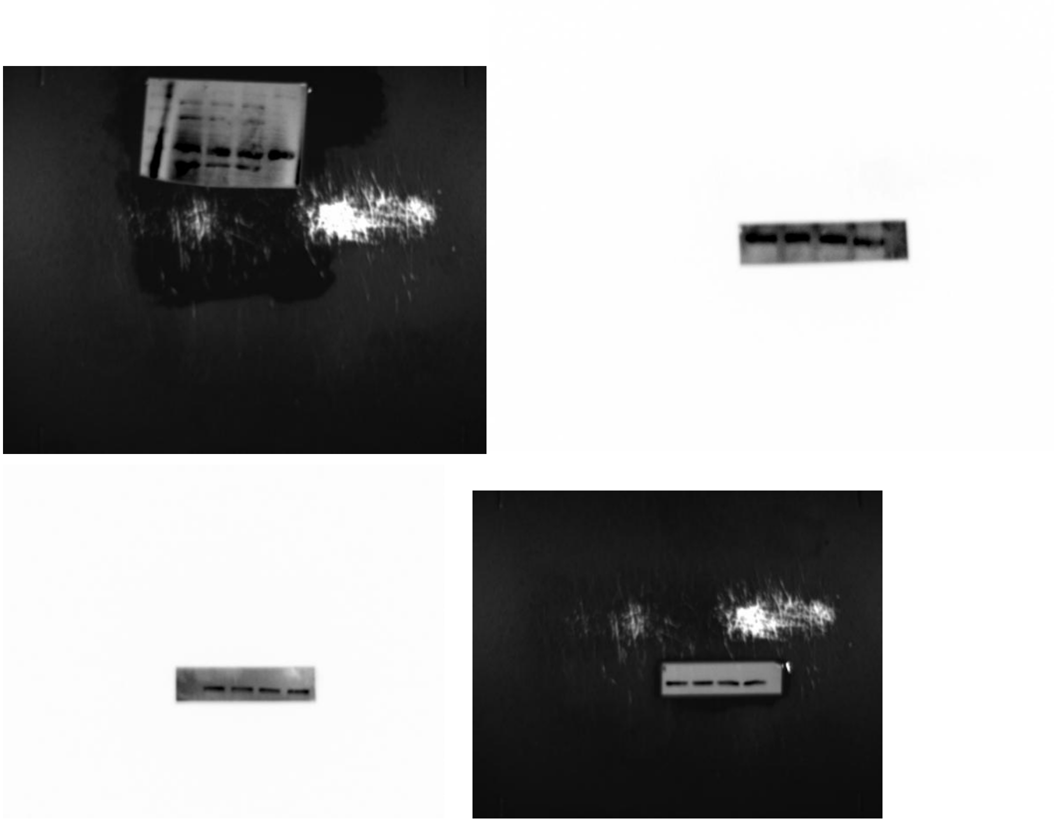

图 I

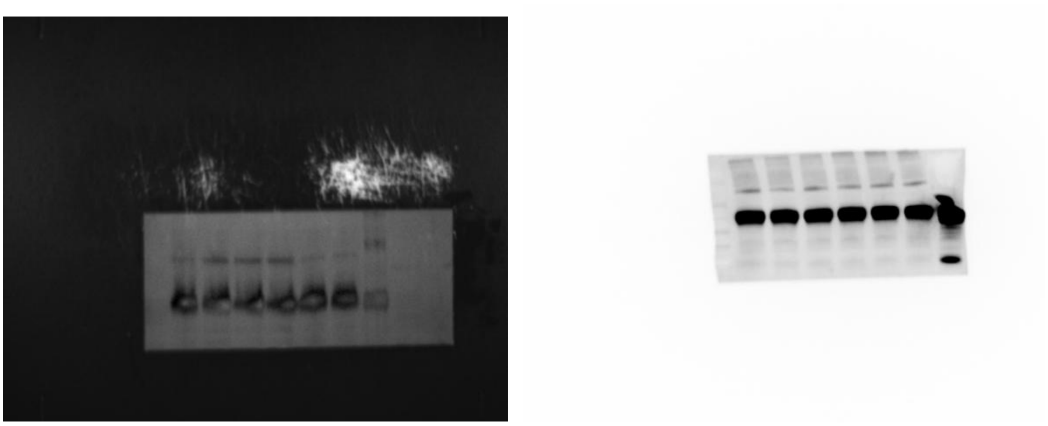

图 J

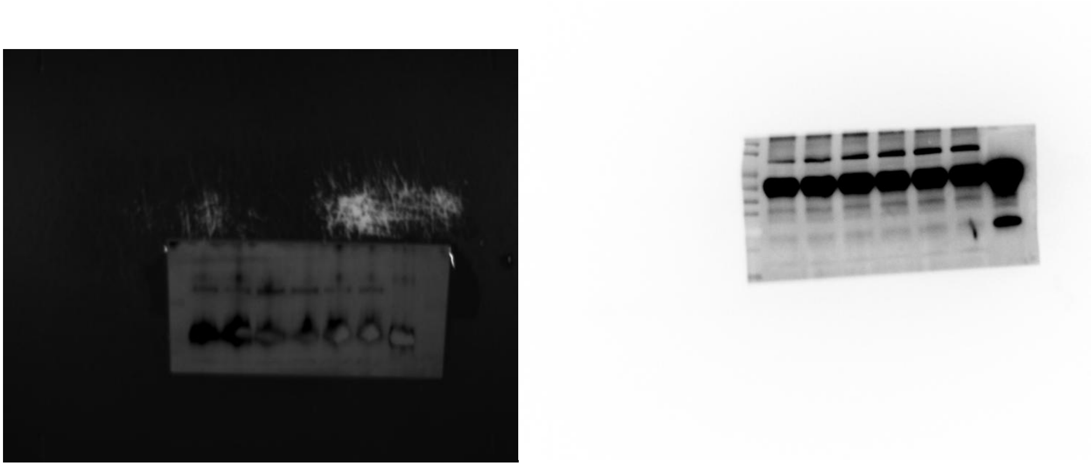

图 9

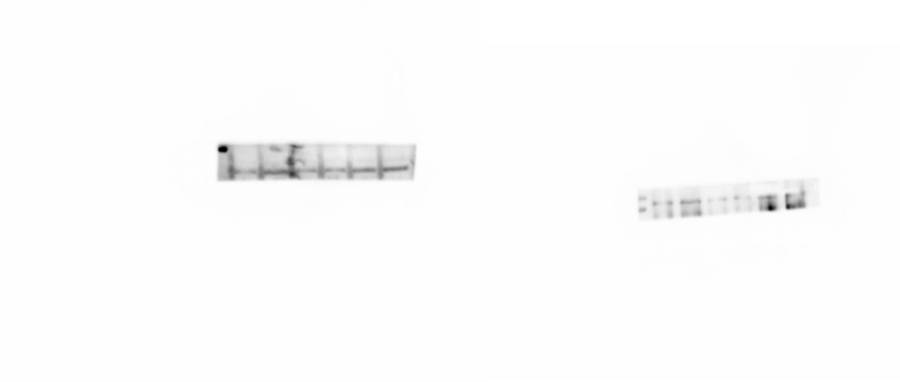

PINK1 PARKIN

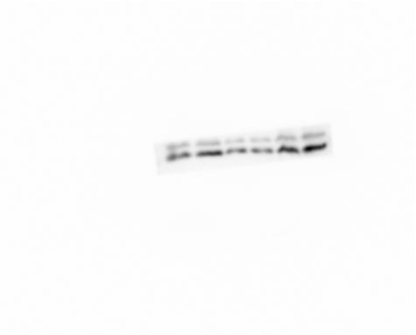
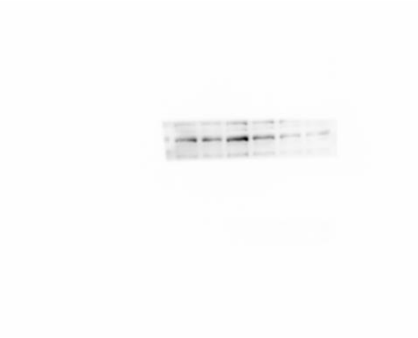

Lc3 P62

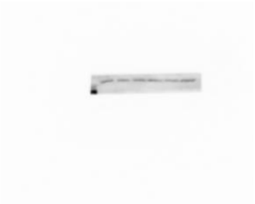

Cox

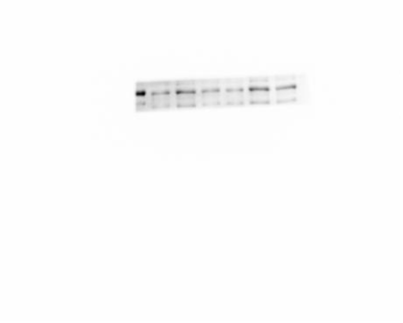
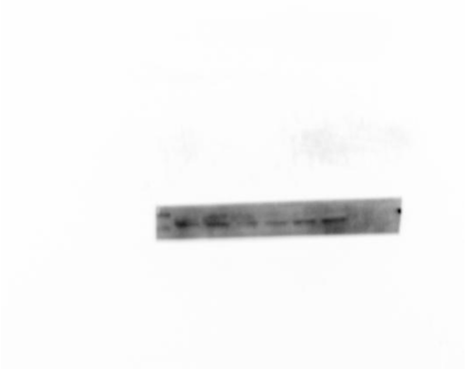

PINK1 Parkin

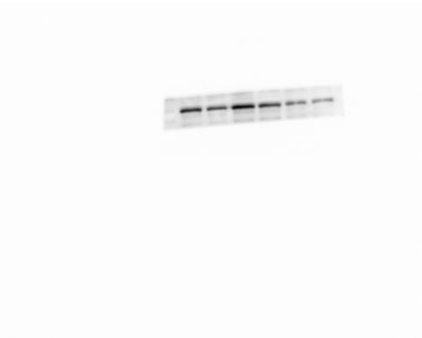
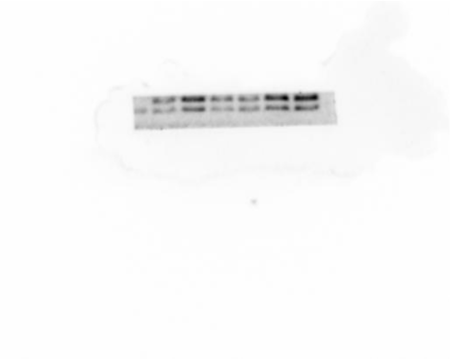

P62 LC3

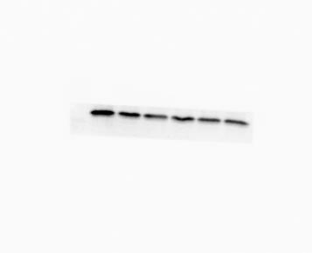

Cox IV
