# Supplementary material for: FGF21‐Mediated Upregulation of SIRT1 Delays Intervertebral Disc Degeneration by Promoting PINK1/Parkin Dependent Mitophagy Through Deacetylation of FOXO3
Source: Aging Cell. 2026 Mar 20;25(4):e70449. doi: 10.1111/acel.70449 (PMC13092504; doi:10.1111/acel.70449)
Supplement: Supplementary file 1 — Appendix S1: acel70449‐sup‐0001‐AppendixS1.docx. [file ACEL-25-e70449-s001.docx]

**Supplementary Materials**

**1 Materials and Methods**

**1.1 Materials**

| Antibody Name | Company | Application and Dilution | Catalog Number |
| --- | --- | --- | --- |
| FGF21 | Abconel | Western Blot（1:1000), IHC (1:200), IF (1:200) | A23463 |
| p16 | Immunoway | Western Blot（1:1000), IHC (1:200), IF (1:200) | YM0494 |
| P21 | Immunoway | Western Blot（1:1000), IHC (1:200), IF (1:20) | YT mitophagy3497 |
| p21 | Abclonel | Western Blot（1:1000) | A19094 |
| p53 | Immunoway | Western Blot（1:1000) | YT3528 |
| parkin | CST | Western Blot（1:1000) | 4211 |
| parkin | Santa | IF (1:50) | sc-32282 |
| PINK1 | Immunoway | Western Blot（1:1000) | YN2037 |
| P62 | Proteintech | Western Blot（1:5000), IHC (1:500), IF (1:500) | 18420-1-AP |
| P62 | Abmart | Western Blot（1:1000) | T55546 |
| LC3 | Proteintech | IHC (1:200), IF (1:500) | 14600-1-AP |
| LC3 | Huaan | Western Blot（1:1000) | ET1701-65 |
| LC3 | Abmart | Western Blot（1:1000) | T55992 |
| LC3 | Santa | IF (1:50) | sc-398822 |
| Tom20 | Proteintech | IF (1:200) | 11801-1-AP |
| Foxo3A | CST | Western Blot（1:1000), IP (1:100) | 8469 |
| Sirt1 | CST | Western Blot（1:1000), IP (1:100), IHC (1:200), IF (1:100) | 2497 |
| Anti-Acetyllysine | PTM BIO | Western Blot（1:1000) | PTM-101 |
| Anti-Acetyllysine | PTM BIO | Western Blot（1:1000), IP (1:50) | PTM-105RM |
| mouse Ig G | Biodragon | IP（1:100） | BF01006 |
| Rabbit Ig G | Biodragon | IP（1:100） | RM4153 |
| GAPDH | Immunoway | Western Blot（1:5000) | YM3029 |
| COX IV | Abmart | Western Blot（1:1000) | T40110 |

**1.2 Experimental Design**

In vitro experiments were conducted to determine the protective effect of FGF-21 on NPCs by treating them with different concentrations of FGF-21 (50, 200 ng/mL) for 24 hours, followed by treatment with TBHP (20 µM). In addition, treatment of TBHP induced myeloid cells with FGF21 was performed to determine the role of FGF21 in autophagic flux activation. Before administering FGF21, Midiv-1(5uM) pretreatment was performed for 2 hours, followed by co treatment with FGF21 for 24 hours to confirm the protective effect of FGF21 on mitophagy activation. To investigate the effect of FGF21 on autophagic flux, bafilomycin A1 (20 nM) was first pretreated for 6 hours, followed by FGF21 treatment for 24 hours.

**1.3 Bioinformatics Analysis**

A total of 8 datasets from the Gene Expression Omnibus (GEO) database were included, namely GSE160756, GSE165722, GSE199866, GSE205535, GSE230809, GSE233666, GSE244889, and GSE251686, along with relevant data from the CNGB Project database. Single-cell RNA sequencing data were normalized and integrated using Seurat v4.0.6 in R v4.2.0. All datasets were first merged into a unified Seurat object and subjected to consistent quality control procedures. Quality control criteria included the removal of cells with mitochondrial gene content exceeding 10%, as well as cells with fewer than 200 or more than 6000 detected genes, to minimize the impact of low-quality cells or potential doublets. Data normalization was performed using the NormalizeData() function with the LogNormalize method, and expression values were scaled using a factor of 10,000 (scale.factor = 10000). Highly variable genes (top 2,000) were identified using the FindVariableFeatures() function with the "vst" method to enhance sensitivity in downstream analyses. The data were then standardized using ScaleData() and dimensionality reduction was performed using principal component analysis (PCA) via the RunPCA() function (npcs = 50). To account for batch effects across different datasets, the Harmony algorithm was used to correct for technical variation and align data across a shared embedding space. Following batch correction and dimensionality reduction, K-nearest neighbor (KNN) clustering was carried out using Seurat’s FindNeighbors() and FindClusters() functions, with the clustering resolution set to 0.5 for optimization using the Louvain algorithm. UMAP visualization was then performed using RunUMAP() (dims = 1:30) to ensure interpretability of the results. Cell type annotation was conducted based on published intervertebral disc (IVD)-related literature and well-established marker genes. Differential gene expression for each cluster was computed using FindAllMarkers(), and annotations were further validated using the SingleR reference dataset. Key marker genes were visualized using DotPlot() and FeaturePlot(), and cells were ultimately classified into degenerative NPCs (NPCs_IDD) and normal NPCs (NPCs_Normal). After identifying the cellular subpopulations, differential expression analysis between degenerative and non-degenerative NPCs was performed using the FindMarkers() function, with selection thresholds set at |log2FC| > 0.25 and adjusted p-value < 0.05. The resulting differentially expressed genes were intersected with aging-related gene sets obtained from the CellAge and GSAE_Senescence_gene databases to identify key genes closely associated with cellular senescence.

**1.4 RNA extraction and reverse transcription quantitative PCR (RT-qPCR)**

Total RNA was extracted from tissues and cells using TRIzol(AG21101; ACCURATE BIOLOGY AG, Guangzhou, China). RNA was reverse transcribed into cDNA using the Evo M-MLV Reverse Transcription Kit (AG11705; ACCURATE BIOLOGY AG). cDNA was amplified using SYBR Premix Ex Taq (AG11001; ACCURATE BIOLOGY AG). The expression of target genes was quantitatively analyzed using the 2^− ΔΔCt method.

Primer Sequences

| Gene Name | Species | Sequence |
| --- | --- | --- |
| FGF21 | Homo | F: GCCTTGAAGCCGGGGGTTATT  R: GTGGAGCGATCCATACAGGG |
| P16 | Homo | F: TGAGCACTCACGCCCTAAGC  R: GTGACTCAAGAGAAGCCAGTAACC |
| P21 | Homo | F: GTCCAGCGACCTTCCTCATCCC  R: GTCCAGCGACCTTCCTCATCC |
| P53 | Homo | F: CGTACTCCCCTGCCCTCAAC  R: ATGTGCTGTGACTGCTTGTAGATG |
| P62 | Homo | F: GTACCAGGACAGCGAGAGGAAG  R: GCAGACACCGAGGAGATGAGG |
| LC3  PINK1  Parkin  BNIP3  BNIP3L  FUNDC1  SIRT1  GAPDH | Homo  Rat  Rat  Rat  Rat  Rat  Rat  Rat | F: ACACACTCCCTTCTGCCTTCC  R: TCTGATGGCTGTCTGACCTAAGAG  F: GAGGAGAAGCAGGCGGAAGAG  R: TCGTGTGTCCAGTGGGTCAG  F: CACAGACAAGGACACATCAGTAGC  R: GGCGGTGGTTACATTGGAAGAC  F: GATGCGCAGCATGAATCTGG  R: CTGAGAGTAGCTGTGCGCTT  F: CTCACTTAGTCGAGCCGCC  R: GCCAACACGTGAGAGAGGAA  F: AGTAATGGGTGGCGTGACTG  R: GCAAAAAGCCTCCCACGAAG  F: ACGCCTTATCCTCTAGTTCCTGTG  R: GGTCTGTCAGCATCATCTTCCAAG  F: AGTAATGGGTGGCGTGACTG  R: GCAAAAAGCCTCCCACGAAG |
| GAPDH | Homo | F: TCAAGAAGGTGGTGAAGCAGG  R: TCAAAGGTGGAGGAGTGGGT |

**1.5 CKK-8 assay**

The Cell Counting Kit-8 (CCK-8) assay (Dojindo, Tokyo, Japan) was performed as per the manufacturer’s instructions. Briefly, NP cells were seeded into a 96-well plate at 1000 cells/well, and were stimulated with IL-1β or PBS and incubated for 0, 12, 24, or 48 h. Next, 10 μL of CCK-8 solution was added to each well, and the cells were incubated at 37 °C in a 5% CO2 incubator for 2 h. Finally, the absorbance was measured at 450 nm.

**1.6 Immunohistochemical staining**

The IVD tissue was embedded in paraffin, cut into 4-µm sections, deparaffinized with xylene, rehydrated using an alcohol gradient, and incubated in sodium citrate (10 mM, 100 °C) and 3% H2O2. The sections were blocked with 10% goat serum and then incubated with primary antibodies overnight at 4 °C. Then, the sections were incubated with biotinylated secondary antibodies and counterstained with hematoxylin. Images were acquired using a light microscope (Olympus Inc., Tokyo, Japan), and the number of positive cells was counted.

**1.7 Histological staining**

The rat tail vertebrae (Co7/8 discs) were fixed with 4% paraformaldehyde for 48 h, decalcified for 3 weeks, embedded in paraffin, and then cut into 4-μm-thick sections. Paraffin sections were stained with hematoxylin and eosin (H&E) and safranin-O staining to observe cell and tissue morphology.

**1.8 Senescence-Associated β-Galactosidase Staining**

According to the instructions of the manufacturer, the Senescence-associated β-Galactosidase (SA-β-gal) Staining Kit (Beyotime, Shanghai, China) was used to assay senescence. NP cells were fixed at room temperature for 15 min and washed twice with PBS. Next, we added 1 mL of β-galactosidase staining solution (10 μL of staining solution A, 10 μL of staining solution B, 930 μL of staining solution C, and 50 μL of X-Gal solution) to the wells and transferred the plates to an incubator (no CO2) at 37°C at least overnight. Under an inverted light microscope (EVOS-FL Cell Imaging System, Thermo Fisher Scientific), aged NP cells with high SA-β-gal activity were blue.

**1.9 Immunofluorescence**

A total of 1 × 106 cells were seeded on coverslips in a six-well plate. Cells were fixed with 4% neutral paraformaldehyde for 20 min, washed three times in PBS, and permeabilised in 0.25% Triton X-100 for 5 min. The cells were washed three times with PBS and blocked (10% goat serum) for 1 h. Next, the cells were incubated with the primary antibody overnight at 4°C followed by a fluorescein isothiocyanate- or cyanine 3-conjugated secondary antibody at 37°C for 1 h. The cells were rinsed three times in PBS for 5 min each, 4′,6-diamidino-2-phenylindole (DAPI, Invitrogen) was applied to counterstain nuclei, and the cells were incubated for 10 min with a chromogen. Finally, the cells were photographed under a fluorescence microscope (Olympus Inc., Tokyo, Japan).

**1.10 Detection of SOD and MDA**

Serum levels of Superoxide Dismutase (SOD) and Malondialdehyde (MDA) were measured using ELISA kits from Beyotime Biotechnology (Shanghai, China), with product codes S0101S and S0131S0, respectively.

**1.11 Measurement of Mitochondrial membrane potential**

Mitochondrial membrane potential (MMP) was measured using the JC-1 Detection Kit (MX3204; MKBio Biotechnology Co., Shanghai, China). In healthy mitochondria, JC-1 forms aggregates in the mitochondrial matrix, emitting red fluorescence. When the MMP decreases, JC-1 appears as monomers in the cytoplasm, producing green fluorescence. Prior to fluorescence imaging, cells were incubated with JC-1 staining working solution (37°C, 20min).

**1.12 Detection of ROS**

Cellular ROS levels were determined using the ROS Detection Kit (MKBio Biotechnology Co., Shanghai, China) with the DCFH-DA probe. Mitochondrial ROS levels were measured with the MitoSOX Red Staining Kit (MCE, USA). Fluorescence microscopy (Olympus BX60, Tokyo, Japan) was used to evaluate staining.

**1.13 Determination of ATP levels**

ATP levels was assessed using the ATP Detection Kit (Nanjing Jiancheng Bioengineering Institute, China) according to the manufacturer’s protocol. The assay utilized the molybdenum blue colorimetric method, and ATP levels was evaluated by the standard curve.

**1.14 Mitochondrial Isolation**

After enzymatic digestion, NPCs were centrifuged at 200 × g for 10 minutes, and the supernatant was discarded. The cell pellet was gently resuspended in pre-chilled PBS and centrifuged at 600 × g for 5 minutes. The supernatant was discarded again, and the cells were resuspended in an appropriate volume of PMSF-containing buffer. The suspension was incubated on ice for 10 minutes and homogenized. The homogenate was then centrifuged at 11,000 × g for 10 minutes to collect the purified mitochondria.

**1.15 X-ray and MRI Examination**

Eight weeks postoperatively, X-ray and MRI imaging of the rat tail was performed. For X-ray imaging, rats were positioned in a horizontal supine posture with the tail extended. Radiographs were taken at a distance of 66 cm between the collimator and the film, using a voltage of 35 kV and a current of 63 mA. For MRI, sagittal T2-weighted images were obtained using a 3.0 T clinical MRI scanner with a fast spin-echo sequence. Imaging parameters included a repetition time (TR) of 5400 ms, echo time (TE) of 920 ms, matrix size of 320(h) × 256(v), field of view (FOV) of 260°, and four signal averages (NEX = 4). The slice thickness was 2 mm with no interslice gap. After completion of X-ray and MRI examinations, rats were euthanized, and the acquired images were subjected to further analysis。

**1.16 Lentivirus transfection**

Lentivirus-coated sh-SIRT1 and sh-NC were purchased from Genechem, Inc. (Shanghai, China). Cells were plated onto a 6-well plate at 1 × 106 cells/well, cultured to 30–50% confluency, and then infected with lentivirus-coated sh-SIRT1 or sh-NC for 12 h. Transfection was performed with lentiviruses at a multiplicity of infection of 100. The culture medium was changed after 12 h of transfection, and the cells were cultured in complete medium for 3–5 days. After the cells reached ~ 80% confluence, they were passaged at 1:3. Transfection efficiency was confirmed using polymerase chain reaction (PCR) and western blotting analysis.

Knockdown Sequences Lists

| Sequence | Target Gene | Oligo-Sequence |
| --- | --- | --- |
| 1 | SIRT1 | GCCACCAACACCTCTTCATAT |
| 2 | SIRT1 | GCAGATTAGTAAGCGTCTTGA |
| 3 | SIRT1 | GGTTCCTTTGCAACAGCATCT |
| 4 | PINK1 | GCTGCAATGCCGCTGTGTA |
| 5 | Parkin | AACGATACTCTGTTGTTCCAG |

**2 Results**

**Table S2** Multifactorial Logistic

| **Variable** | **β** | **SE** | **Wald** | **OR** | **95%CR** | ***P value*** |
| --- | --- | --- | --- | --- | --- | --- |
| age | 0.082 | 0.061 | 1.788 | 1.085 | 0.963 1.223 | 0.181 |
| Symptom.Duration | 1.842 | 1.308 | 1.983 | 6.309 | 0.486 81.915 | 0.159 |
| FGF21.IHC.Score | -0.506 | 0.489 | 1.069 | 0.603 | 0.231 1.573 | 0.301 |

Supplementary Fig 1 FGF21 Is Downregulated During IDD. (A) Hematoxylin and eosin (H&E) staining of human nucleus pulposus (NP) tissues. (B) Quantitative analysis of proteoglycan content. (C) Quantitative analysis of immunohistochemical staining of Aggrecan in human NP tissues. (D) Quantification of senescence-related protein expression levels in human NP tissues by Western blot. (E–G) Quantitative analysis of immunohistochemical staining for P16, P21, and FGF21 in human NP tissues. (H–I) Statistical bar charts of Pfirrmann grades and disc height index (DHI) in rat caudal intervertebral discs. (J) Heatmap of histological scores in rat intervertebral discs. (K) CCK assay of NPCs treated with TBHP across different time points and concentrations. (L–M) Western blot analysis and quantification of senescence-related protein expression in NPCs following TBHP treatment over a time gradient. (N) Quantification of SA-β-Gal-positive cells. (O) Intracellular ATP levels measured by colorimetric assay. (P) Fluorescence images of mitochondrial membrane potential assessed using JC-1 staining. (Q)Representative transmission electron microscopy (TEM) images. Black arrows indicate lysosomes, white arrows indicate mitochondria, yellow arrows indicate vacuolated mitochondria, green arrows indicate autophagosomes, blue arrows indicate autophagosome, and red arrows indicate autolysosomes.

Supplementary Fig 2 Sankey Diagram Showing the Correlation Between FGF21 Expression and Clinicopathological Features.

Supplementary Fig 3 FGF21 Inhibits NPC Senescence. (A) CCK-8 assay showing the effect of FGF21 on cell viability.. (B) Quantification bar graph of mitochondrial membrane potential. (C) Colorimetric assay measuring cellular MDA levels. (D) Quantification bar graph of intracellular reactive oxygen species (ROS) levels. (E) Quantification bar graph of mitochondrial ROS levels.

Supplementary Fig4 FGF21 attenuates senescence in NPCs. (A–B) Western blot analysis of senescence-related protein expression in NPCs treated with varying concentrations of FGF21, along with corresponding quantitative bar graphs. (C–D) Representative images and quantification of SA-β-Gal staining showing the effect of FGF21 treatment on NPCs senescence. (E) Fluorescence images of mitochondrial membrane potential assessed using JC-1 staining. (F) Intracellular ATP levels measured by colorimetric assay. (G) Colorimetric assay measuring cellular MDA levels. (H) Representative fluorescence images of intracellular reactive oxygen species (ROS) detected with ROS probes. (H–I) Representative images and quantitative analysis of mitochondrial ROS levels detected using MitoSOX staining. The quantitative analysis of immunofluorescence images is based on randomly selecting 5 fields of view from each biological replicate and taking the average, and conducting at least six independent experiments. The data points in the report represent the combined measurement values of all analyzed cells.

Supplementary Fig 5 FGF21 Activates mitophagy. (A) Volcano plot of differentially expressed genes. (B) GSEA enrichment analysis of differentially expressed genes.

Supplementary Fig 6 Examine the effects of TBHP (A–B) and FGF21 (C–D) on autophagy in NPCs in the presence or absence of bafilomycin A1.

Supplementary Fig 7 FGF21 Activates mitophagy. (A–B) Quantitative bar graphs of LC3 and P62 immunohistochemical staining in human intervertebral disc nucleus pulposus tissue. (C–D) Quantitative bar graphs of LC3 and P62 immunohistochemical staining in rat coccygeal intervertebral discs. (E) Quantitative bar graph of mCherry-EGFP-LC3 dual-labeled adenovirus for autophagy. (F) Quantitative bar graph from transmission electron microscopy.

Supplementary Fig8 Midiv-1 abrogated the FGF21-induced activation of mitophagy (A) The CKK-8 assay was used to detect the cell viability of NPCs after 24 hours of intervention with different concentrations of Midiv-1. (B) The CKK-8 assay was used to detect the cell viability of NPCs after intervention with 5uM Midiv-1 for 6, 12, 24 and 48 hours. (C-E) Western blot analysis and quantification of LC3 and P62 in mitochondrial fractions. (F) Representative confocal images showing colocalization of LC3 with mitochondrial outer membrane protein Tom20. (G-H) Confocal microscopy images of cells transduced with tandem mCherry-EGFP-LC3 virus, indicating autophagosomes (yellow puncta) and autolysosomes (red puncta). The quantitative analysis of immunofluorescence images is based on randomly selecting 5 fields of view from each biological replicate and taking the average, and conducting at least six independent experiments. The data points in the report represent the combined measurement values of all analyzed cells.

Supplementary Fig 9 Drp-1 knockdown reversed the inhibitory effect of FGF21 on NPCs senescence. (A) qPCR analysis of Drp-1 mRNA expression levels. (B–C) Western blot analysis and corresponding quantification bar graphs of Drp-1 protein levels. (D-E) Western blot analysis and quantification of P53, P21, and P16 protein expression. (F-G) Representative images and quantification of SA-β-Gal staining to assess cell senescence. (H) JC-1 staining for mitochondrial membrane potential. (I) Colorimetric assay to determine intracellular ATP levels.(J) Fluorescence imaging of intracellular ROS using DCFH-DA probe and of mitochondrial ROS levels assessed using Mito-Sox probe. The quantitative analysis of immunofluorescence images is based on randomly selecting 5 fields of view from each biological replicate and taking the average, and conducting at least six independent experiments. The data points in the report represent the combined measurement values of all analyzed cells.

Supplementary Fig10 Drp-1 knockdown suppressed FGF21-induced activation of mitophagy. (A-C) Western blot analysis and quantification of LC3 and P62 in mitochondrial fractions. (D) Representative confocal images showing colocalization of LC3 with mitochondrial outer membrane protein Tom20. (E-F) Confocal microscopy images of cells transduced with tandem mCherry-EGFP-LC3 virus, indicating autophagosomes (yellow puncta) and autolysosomes (red puncta). The quantitative analysis of immunofluorescence images is based on randomly selecting 5 fields of view from each biological replicate and taking the average, and conducting at least six independent experiments. The data points in the report represent the combined measurement values of all analyzed cells.

Supplementary Fig11. FGF21 Activates PINK1/Parkin-Mediated mitophagy. (A) qPCR analysis of genes related to the mitophagy pathway. (B–D) Western blot analysis and corresponding quantification bar graphs of total cellular PINK1, Parkin, p-PINK1 and p-Parkin protein expression in whole-cell lysate. (E–H) Western blot analysis and corresponding quantification bar graphs of PINK1 and Parkin expression in the cytoplasm and mitochondria. (I) Immunofluorescence images showing co-localization of Parkin with the mitochondrial membrane protein Tom20. The quantitative analysis of immunofluorescence images is based on randomly selecting 5 fields of view from each biological replicate and taking the average, and conducting at least six independent experiments. The data points in the report represent the combined measurement values of all analyzed cells.

Supplementary Fig 12. FGF21 Activates the PINK1-Parkin Pathway to Inhibit Nucleus Pulposus Cell Senescence. (A) qPCR analysis of PINK1 and Parkin mRNA expression levels. (B–C) Western blot analysis and corresponding quantification bar graphs of PINK1 and Parkin protein levels. (D)Colorimetric assay of intracellular ATP levels. (E–F) JC-1 probe detection of mitochondrial membrane potential. (G) WST-8 assay to measure intracellular antioxidant SOD levels. (H) Colorimetric assay to measure intracellular MDA levels. (I) DCFH-DA probe detection of intracellular ROS levels. (J) MitoSOX probe detection of mitochondrial ROS levels. (K–L) Quantification of intracellular and mitochondrial ROS. The quantitative analysis of immunofluorescence images is based on randomly selecting 5 fields of view from each biological replicate and taking the average, and conducting at least six independent experiments. The data points in the report represent the combined measurement values of all analyzed cells.

Supplementary Fig13. FGF21 Activates PINK1/Parkin-Mediated mitophagy.(A–B) Western blot analysis and corresponding quantification bar graphs of total cellular SIRT1, LC3, P62, PINK1, Parkin, p-PINK1 and p-Parkin protein expression in whole-cell lysate. (C–D) Western blot analysis and corresponding quantification bar graphs of LC3, P62, PINK1 and Parkin expression in the cytoplasm. (E–F) Western blot analysis and corresponding quantification bar graphs of LC3, P62, PINK1 and Parkin expression in mitochondria. (G-H) The mitochondrial fraction ratio of PINK1and Parkin. (I) Immunofluorescence images showing co-localization of LC3 with the mitochondrial membrane protein Tom20. (J) Immunofluorescence images showing co-localization of Parkin with the mitochondrial membrane protein Tom20. The quantitative analysis of immunofluorescence images is based on randomly selecting 5 fields of view from each biological replicate and taking the average, and conducting at least six independent experiments. The data points in the report represent the combined measurement values of all analyzed cells.

Supplementary Fig 14. SIRT1 Overexpression Inhibits NPC Senescence. (A-B) Western blot analysis and corresponding quantification bar graph of senescence-related protein expression. (C-D) Representative images and quantification bar graph of SA-β-gal staining to assess NPC senescence. (E-F) JC-1 probe fluorescence images and quantification bar graph of mitochondrial membrane potential. (G) Colorimetric assay of intracellular ATP levels. (H) WST-8 assay to measure intracellular antioxidant SOD levels. (I-K) Representative images and quantification bar graph of intracellular ROS detected by ROS probe. (J-L) Representative images and quantification bar graph of mitochondrial ROS detected by mito-ROS probe. (M) Colorimetric assay of intracellular MDA levels. The quantitative analysis of immunofluorescence images is based on randomly selecting 5 fields of view from each biological replicate and taking the average, and conducting at least six independent experiments. The data points in the report represent the combined measurement values of all analyzed cells.

Supplementary Fig 15. Overexpression of SIRT1 activates mitophagy via the PINK1-Parkin pathway. (A–B) Western blot analysis and corresponding quantification bar graphs of total cellular PINK1, Parkin, p-PINK1 and p-Parkin protein expression in whole-cell lysate. (C–D) Western blot analysis and corresponding quantification bar graphs of PINK1 and Parkin expression in the cytoplasm. (E–F) Western blot analysis and corresponding quantification bar graphs of PINK1 and Parkin expression in mitochondria. (G) The mitochondrial fraction ratio of PINK1and Parkin. (H) Immunofluorescence images showing co-localization of LC3 with the mitochondrial marker Tom20. (I) Immunofluorescence images showing co-localization of Parkin with Tom20. The quantitative analysis of immunofluorescence images is based on randomly selecting 5 fields of view from each biological replicate and taking the average, and conducting at least six independent experiments. The data points in the report represent the combined measurement values of all analyzed cells.

Supplementary Fig 16. FGF21 Inhibits NPC Senescence by Upregulating SIRT1. (A) qPCR analysis of Sirt mRNA expression. (B–C) Western blot analysis and corresponding quantification bar graphs of SIRT1 protein expression levels. (D–E) Western blot analysis and quantification bar graphs of senescence-related protein expression. (F–G) Representative images and quantification bar graph of SA-β-gal staining to assess NPC senescence. (H) Colorimetric assay of intracellular ATP levels. (I–J) JC-1 probe fluorescence images and quantification bar graph of mitochondrial membrane potential. (K) WST-8 assay to measure intracellular antioxidant SOD levels(L) Colorimetric assay of intracellular MDA levels. (M) Representative images of intracellular ROS detected by ROS probe and mitochondrial ROS detected by mito-ROS probe. The quantitative analysis of immunofluorescence images is based on randomly selecting 5 fields of view from each biological replicate and taking the average, and conducting at least six independent experiments. The data points in the report represent the combined measurement values of all analyzed cells.

Supplementary Fig 17. (A) Quantitative bar graph of Western blot detection of PINK1, Parkin, p-PINK1, and p-Parkin protein expression in whole-cell lysates. (B) Quantitative bar graph of Western blot detection of PINK1, Parkin, p-PINK1, and p-Parkin protein expression in the cytoplasm. (C) Quantitative bar graph of Western blot detection of PINK1, Parkin, p-PINK1, and p-Parkin protein expression in mitochondria. (D) The mitochondrial fraction ratio of PINK1and Parkin. (E)Quantitative bar graphs of representative confocal microscopy images of cells infected with mCherry-EGFP-LC3 dual-labeled autophagic adenovirus. (F) Quantitative bar graphs of transmission electron microscopy (TEM) images.

Supplementary Fig 18. Functional Analysis of Differentially Expressed Genes After SIRT1 Knockdown in NPCs. (A) Heatmap of differentially expressed genes. (B) GSEA pathway enrichment analysis of differentially expressed genes.

Supplementary Fig 19. Identification of SIRT1-Interacting Proteins. (A) Workflow diagram of co-IP/MS analysis. (B) Coomassie brilliant blue staining. (C) GO enrichment analysis. (D) KEGG enrichment analysis. (E) PPI network. (F) FOXO3 identified as an immunoprecipitated product from anti-SIRT1 antibody co-IP/MS. (G) Conservation of FOXO3 protein sequence across species.

Supplementary Fig 20 The deacetylation of FOXO3 is crucial for FGF21 to protect NPCs from TBHP-induced cellular senescence. (A) Representative Western blot images and quantification bar graphs of P16, P21 and P53 expression in NPCs with or without FGF21. (B) Representative images and quantitative bar graphs assessing the cell senescence, with or without FGF21 intervention on NPCs, using SA-β-gal staining. (C) Representative Western blot images and quantification bar graphs of P16, P21 and P53 expression in NPCs with or without SIRT1 overexpression. (D Representative images and quantitative bar graphs assessing the cell senescence, with or without SIRT1 overexpression on NPCs, using SA-β-gal staining. The quantitative analysis of SA-β-gal staining images is based on randomly selecting 5 fields of view from each biological replicate and taking the average, and conducting at least six independent experiments. The data points in the report represent the combined measurement values of all analyzed cells

Supplementary Fig 21 FGF21 Promotes mitophagy in NPCs via SIRT1-Mediated Deacetylation and Activation of FOXO3. (A) Representative Western blot images and corresponding quantification bar graphs of PINK1, Parkin, LC3-II, and P62 protein expression in NPCs with or without SIRT1 knockdown. (B) Confocal microscopy imaging and quantification bar graphs of live NPCs with or without SIRT1 knockdown (red puncta indicate autophagosomes; red-only puncta indicate autolysosomes). The quantitative analysis of immunofluorescence images is based on randomly selecting 5 fields of view from each biological replicate and taking the average, and conducting at least six independent experiments. The data points in the report represent the combined measurement values of all analyzed cells.

Supplementary Fig 22 FGF21 inhibits the senescence of NPCs by mediating the deacetylation of FOXO3 through SIRT1. (A) Representative Western blot images and quantification bar graphs of P16, P21 and P53 expression in NPCs with or without SIRT1 knockdown. (B) Representative images and quantitative bar graphs assessing the cell senescence, with or withoutSIRT1 knockdown on NPCs, using SA-β-gal staining. The quantitative analysis of SA-β-gal staining images is based on randomly selecting 5 fields of view from each biological replicate and taking the average, and conducting at least six independent experiments. The data points in the report represent the combined measurement values of all analyzed cells
